# STAT3/LINC00671 axis regulates papillary thyroid tumor growth and metastasis via LDHA-mediated glycolysis

**DOI:** 10.1038/s41419-021-04081-0

**Published:** 2021-08-17

**Authors:** Nan Huo, Rui Cong, Zhi-jia Sun, Wen-chao Li, Xiang Zhu, Chun-yuan Xue, Zhao Chen, Lu-yuan Ma, Zhong Chu, Yu-chen Han, Xiao-feng Kang, Song-hao Jia, Nan Du, Lei Kang, Xiao-jie Xu

**Affiliations:** 1 Department of Genetic Engineering, Beijing Institute of Biotechnology, Beijing, China; 2 Department of Oncology, Fourth Medical Center of PLA General Hospital, Beijing, China; 3 Department of Paediatric Orthopaedic Surgery, Seventh Medical Center of PLA General Hospital, Beijing, China; 4 Department of Nuclear Medicine, Peking University First Hospital, Beijing, China

**Keywords:** Oncogenes, Cancer metabolism

## Abstract

Lactate dehydrogenase A (LDHA), a critical component of the glycolytic pathway, relates to the development of various cancers, including thyroid cancer. However, the regulatory mechanism of LDHA inhibition and the physiological significance of the LDHA inhibitors in papillary thyroid cancer (PTC) are unknown. Long non-coding RNA (lncRNA) plays a vital role in tumor growth and progression. Here, we identified a novel lncRNA LINC00671 negatively correlated with LDHA, downregulating LDHA expression and predicting good clinical outcome in thyroid cancer. Moreover, hypoxia inhibits LINC00671 expression and activates LDHA expression largely through transcriptional factor STAT3. STAT3/LINC00671/LDHA axis regulates thyroid cancer glycolysis, growth, and lung metastasis both in vitro and in vivo. In thyroid cancer patients, LINC00671 expression is negatively correlated with LDHA and STAT3 expression. Our work established STAT3/LINC00671/LDHA as a critical axis to regulate PTC growth and progression. Inhibition of LDHA or STAT3 or supplement of LINC00671 could be potential therapeutic strategies in thyroid cancer.

## Introduction

Thyroid cancer (TC) is the most prevalent endocrine malignancy and one of the most rapidly increasing cancers around the world [1]. Depending on histological characteristics, thyroid carcinoma can be classified into five types, among which well-differentiated papillary thyroid cancer (PTC) accounts for the most majority cases [2]. However, 10% of PTC cases dedifferentiate into the aggressive type, characterized by the metastases at early stages to the lungs (50%) and other sites with local invasion and/or distant metastasis [3, 4]. To this end, it is urgent and essential to identify novel treatment targets for PTC and understand the molecular mechanisms during PTC initiation and progression.

Energy metabolism reprogramming is widely accepted as a novel hallmark of cancer, characterized by high glycolysis regardless of the presence of abundant oxygen [5, 6]. This glycolysis process is usually accompanied by glucose uptake and lactate production, as well as ATP generation, facilitating tumor growth and progression. Glycolytic enzymes affecting the critical stages of the process, play vital roles in cancer cell growth and progression, and become potential targets for cancer treatment [7, 8]. LDHA, one of the key enzymes, catalyzes the final step of glycolysis and promotes the efficiency of glycolysis in tumor cells and reduces their dependence on oxygen [9, 10]. LDHA is highly expressed in cancer cells and regarded as a biomarker of multiple malignant cancers, including lymphoma, prostate cancer, renal cell carcinoma, melanoma as well as PTC [7–10]. However, the regulatory mechanisms of LDHA inhibition and the physiological significance of the LDHA inhibitors in PTC are unknown.

Long non-coding RNAs (lncRNAs) are a major class of non-coding RNAs with a length of more than 200 nt, which exerts their function through epigenetic modulation, transcriptional (or post transcriptional), and translational regulation of targeted genes [11]. Accumulating profiling studies have revealed that lncRNAs are aberrantly expressed in various human cancers, and involved in tumor development, progression, and metastasis. In thyroid cancer, several lncRNAs, such as MALAT1, H19, BANCR, and HOTAIR have been identified as contributing factors to tumor development, and used as novel biomarkers for early diagnosis or treatment [12]. However, whether lncRNAs regulate glycolysis in PTC remains largely unknown.

Homo sapiens long intergenic non-protein coding RNA 00671 (LINC00671) is a long non-coding RNA that produces a 1.84 kb transcript. Previous study reported that LINC00671 was downregulated in pancreatic cancerous tissues and serum, and silencing of LINC00671 promoted pancreatic cancer cell proliferation [13]. However, the function of LINC00671 in PTC remains unknown. In this study, we identified LINC00671 as a novel LDHA negatively correlated lncRNA, which predicts good clinical outcome of PTC. Moreover, hypoxia inhibits LINC00671 expression and activates LDHA expression largely through the transcription factor STAT3. STAT3/LINC00671/LDHA axis regulates glycolysis, tumor growth, and lung metastasis of PTC both in vitro and in vivo. Our work establishes STAT3/LINC00671/LDHA as a novel and critical axis regulating PTC growth and progression.

## Materials and methods

### Cell culture, plasmids, lentivirus, RNA oligonucleotides, and reagents

The human thyroid cancer cell line (TPC-1, BCPAP) was obtained from the American Type Culture Collection (Manassas, VA, USA) and tested for mycoplasma contamination. All of these cells were maintained in RPMI-1640 medium supplemented with 100 U/ml penicillin, 100 μg/ml streptomycin, and 10% fetal bovine serum (FBS) at 37 °C in a humidified atmosphere of 5% CO_2_. Cells exposed to hypoxia condition were performed by placing the cells in variable oxygen control (tri-gas) CO_2_ incubator (Thermo Scientific) filled with mixture gases of 1% O_2_, 5% CO_2_, and 94% N_2_ for indicated time. The eukaryotic expression vectors were generated by inserting PCR-amplified fragments into pcDNA3.0 (Invitrogen). The LINC00671 promoter and its mutant luciferase reporters were made by inserting PCR-amplified promoter fragments from genomic DNA into the pGL4.0-Basic vector (Promega, Madison, WI, USA). Wild-type and mutated STAT3 putative targets on LINC00671 were cloned into pGL4.0-Basic vector (Promega). Smart pool of LINC00671 Silencers was synthesized by RiboBio Company. The target sequences of siRNAs and/or shRNAs for LDHA, LINC00671, and STAT3 are listed in Supplementary Table S1. Lentiviral vector expressing LDHA shRNA or STAT3 shRNA was constructed by cloning LDHA shRNA or STAT3 shRNA fragment into pSIH-H1-Puro (System Biosciences). Stable cell lines with LDHA shRNA plus LINC00671 shRNA were generated by infection with the lentiviruses carrying LDHA shRNA and LINC00671 shRNA. The stable cells were selected using puromycin. Lipofectamine 2000 and Lipofectamine RNAiMAX were used for transfections of plasmids and siRNAs according to the manufacturer’s instructions (Invitrogen). Anti-LDHA and HIF-1α were purchased from Proteintech. Anti-STAT3 and Anti-STAT3 (Y705) were purchased from Cell Signaling Technology. Anti-Ki67 was purchased from Servicebio. Niclocide was purchased from Selleck Chemicals.

### RNA extraction and real-time quantitative PCR (qRT-PCR)

Total RNA of cell samples was isolated by using the TRizol reagent (Invitrogen, USA), according to the manufacturer’s protocols. RT-PCR was performed on the CFX96 system (BioRad Laboratories Inc., USA) using SYBR Green. The volume of the reaction system is 20 μl, which contains 10 μl 2 × SYBR Green I, 0.4 μl sense primer, 0.4 μl antisense primer, and 9.2 μl diluted template. The relative expression of different gene sets was normalized to β-actin. The relative folding expression of the target normalized to the corresponding control was calculated by the comparative *Ct* method. The primer sequences used for qRT-PCR are listed in Supplementary Table S2.

### Fluorescence in situ hybridization (FISH)

TPC-1 and BCPAP were washed in PBS, and then fixed in 4% paraformaldehyde for 10 min at room temperature. Cells were permeabilized in 1 × PBS containing 0.5% Triton X-100 for 5 min at 4 °C, then washed in 1 × PBS for 5 min. Two hundred microliters of Pre-hybridization Buffer was added at 37 °C for 30 min. Hybridization was carried out with a FISH probe in a moist chamber at 37 °C in the dark overnight using Ribo^TM^ Fluorescent In Situ Hybridization Kit (C10910, RiboBio). The slides were washed three times with Wash Buffer I (4 × SSC with 0.1% Tween-20), once each with Wash Buffer II (2 × SSC), Wash Buffer III (1 × SSC) at 42 °C in the dark for 5 min and once with 1 × PBS at room temperature. Then the cells were stained with DAPI in the dark for 10 min. LINC00671 FISH probes were designed and synthesized by RiboBio Company (Guangzhou, China). Human U6 FISH probes and 18S FISH probes were used as the nuclear and cytoplasmic internal controls, respectively.

### Lactate acid, glucose uptake, and ATP measurement

Lactate acid assay kit II, glucose uptake colorimetric assay kit, and ATP colorimetric assay kit were used to determine lactate acid, glucose uptake, and ATP levels according to the manufacturer’s protocol (Biovision).

For the lactate acid assay, cells were seeded into 10 cm plates, transfected or infected with the designated constructs, and incubated in RPMI-1640 supplemented with 10% FBS for 48h. Then cells were plated into a 12-well plate and incubated for 10 hours. Then the media were removed and the cells were incubated in RPMI-1640 without FBS. After incubation for 1 hour, the supernatant was collected for measurement of lactate production (Biovision). The reaction mixture was incubated for 30 min at room temperature and protected from light. Lactate level was measured at 450 nm in a microplate reader. The results are normalized to the number of cells. For the glucose uptake assay, cells were transfected or infected as in lactate acid assay. Cells were seeded into a 96-well plate and incubated for 10 h. Currently, the number of cells in each group was remarkably similar. The cells were washed three times with PBS, and then the glucose was started by pre-incubating with 100 μl Krebs-Ringer-phosphate-HEPES (KRPH) buffer containing 2% BSA for 40 min. Ten microliters of 10 mM 2-DG was added and incubated for 20 min. Cells were lysed with 90 μl extraction buffer, then frozen/thawed once and heated at 85 °C for 40 min. The cell lysate was neutralized by adding 10 μl of neutralization buffer. After centrifugation at 12,000 rpm for 5 min, the supernatant was used to determine glucose uptake (Biovision). Glucose uptake was measured at 412 nm in a microplate reader. The results are normalized to the number of cells.

For ATP level analysis, cells were transfected or infected as in lactate acid assay. Cells were collected and extracted in 100 μl of the ATP Assay Buffer (Biovision). The cells were centrifuged at 12,000 rpm for 5 min, and the supernatant was used for ATP determination. The reaction mixture was incubated at room temperature for 30 min, protected from light, and measured at 570 nm in a microplate reader. The value was normalized to the number of cells.

### Extracellular acidification rate and oxygen consumption rate assays

The extracellular acidification rate (ECAR) and cellular oxygen consumption rate (OCR) were measured using the Seahorse XFe^96^ Extracellular Flux Analyzer (Seahorse Bioscience). The experiment was performed according to the manufacturer’s instructions. ECAR and OCR were measured using Seahorse XF glycolysis stress test kit and Seahorse XF cell mitochondrial stress test kit (Agilent Technologies). Briefly, cells were transfected or infected as in glucose uptake assay. The transfected or infected cells were harvested, and the cell number was counted. Then, 10,000 cells per well were seeded in the Seahorse XF^96^ cell culture microtiter plate for 10 h. The number of cells in each group was similar. These cells were used to measure ECAR and OCR. After the baseline measurement, for ECAR, glucose, oxidative phosphorylation inhibitor oligomycin, and glycolysis inhibitor 2-DG were sequentially injected into each well at a specified time point. For OCR, oligomycin, the reversible inhibitor of oxidative phosphorylation FCCP (p-trifluoromethoxy carbonyl cyanide phenylhydrazone), and the mitochondrial complex I inhibitor rotenone plus the mitochondrial complex III inhibitor antimycin A (Rote/AA) were sequentially injected. The data were analyzed by Seahorse XFe^96^ Wave software. The results were normalized to the number of cells.

### Cell growth and colony formation assays

Cell Counting Kit-8 (CCK-8) analysis was performed to determine cell proliferation according to the manufacturer’s instructions (Dojindo, Japan). To perform the colony formation assay, the transfected cells were plated in a 3.5 cm plate (3000 cells/well). Two weeks later, the colonies were fixed with 4% paraformaldehyde for 30 min, and then stained with 1% crystal violet for 30 min. The number of colonies with diameters of more than 1.5 mm was counted.

### Cell migration and invasion analysis

Cell migration was examined by wound healing assays. Transfected cells grown to 90% in 6-well plates were scratched via a 200 µl pipette tip to create the wound followed by washing twice with PBS. The cultured cells were grown for 24 h to allow the wound to close. The wound healing rates were calculated and compared to the width at 0 h.

The cell invasion assay was performed in Matrigel Invasion Chambers according to the manufacturer’s protocol (BD Biosciences). The transfected cells were seeded into the upper well. After 24 h, the invaded cells were fixed with 4% paraformaldehyde and stained with 0.5% crystal violet for 30 min. The number of invasive cells was counted in casually selected microscope visions and photographed.

### Luciferase reporter assay

1 × 10^5^ cells per well were plated in 24-well plates. Cells were co-transfected with luciferase reporters, either LINC00671 or its truncations luciferase reporter constructs containing either wild-type or mutant STAT3 using Lipofectamine 2000. After 48 h, cells were harvested and analyzed for luciferase and β-galactosidase activities according to the manufacturer’s instructions (Promega). Experiments were performed in triplicate and repeated three times.

### Chromatin immunoprecipitation (ChIP) analysis

ChIP analysis was performed using Magna ChIP G Analysis Kit (Millipore) according to the manufacturer’s instructions. By incubating with 10 mM DTT at 37 °C for 30 min and in ChIP buffer (150 mM NaCl, 1% Triton X-100, 2 mM EDTA, 20 mM Tris-HCl, pH 8.1). A promoter pair of specific primer for the LINC00671 promoter was used to amplify the immunoprecipitated DNA. The value was normalized to the value of IgG or empty vector control.

### Cytosolic and nuclear fractionation

The localization of the protein is determined by subcellular classification. Briefly, TPC-1 and BCPAP cells were washed with PBS twice and incubated with lysis buffer (25 mM Tris-HCl, pH 7.4, 1 mM MgCl_2_, 5 mM KCl, and 1% NP-40) on ice for 10 min. Cells were homogenized using a Dounce homogenizer, and the homogenate was centrifuged at 2000 rpm for 10 min. The pellet was analyzed as the nuclear fraction. The supernatant was centrifuged again at 12,000 rpm for 10 min, and then the final supernatant was analyzed as the cytoplasmic fraction.

### In vivo tumor growth and metastasis analysis

The animal study was approved and monitored by the Ethics Committee of Beijing Institute of Biotechnology. For in vivo tumor estimation, a total of 1 × 10^7^ TPC-1 cells with different constructs were inoculated subcutaneously into the right flanks of nude mice (*n* = 8) using random number method with no blinding. The tumor size was calculated and sacrificed at the indicated time. The resected tumor was conserved in liquid nitrogen.

For lung metastasis studies, 1 × 10^6^ TPC-1 cells carrying indicated constructs were injected into the lateral tail vein of BALB/c nude mice. To investigate the lung metastasis, all mice were maintained for about 50 days until sacrifice. All lungs were excised for metastatic foci analysis.

### LncRNA in situ hybridization and immunohistochemistry

Forty-five human thyroid cancer samples were obtained from Beijing Haidian Hospital with the informed consent of Beijing Haidian Hospital. All individuals signed informed consents. Twenty-four patients underwent PET imaging. The formalin-fixed and paraffin-embedded tissue sections were subjected to immunohistochemistry (IHC) and fluorescent in situ hybridization (FISH). For IHC, tissue sections were deparaffinized, rehydrated, and treated with 3% H_2_O_2_ for 15 min to inhibit endogenous peroxidase activity. After heat-induced epitope recovery in 10 mM citrate buffer (pH 6.0) in microwave for 30 min, pre-diluted primary antibodies were incubated overnight at 4 °C [anti-LDHA (1:100) (Proteintech), anti-STAT3 (1:100) (Cell Signaling Biotechnology)]. After incubation with a secondary antibody, the signal was developed with 3,3′-diaminobenzidine tetrachloride. For detection of LINC00671 by FISH, paraffin PTC tissue sections with a specific probe for human LINC00671 were conducted according to the manufacturer’s instructions (RiboBio, Guangzhou, China).

Two pathologists who do not know the information of the patients were asked to independently assess the expression. The LINC00671, STAT3, and LDHA score was generated by multiplying the percentage of stained cells (0–100%) by the intensity of the staining (low, 1+; medium, 2+; strong, 3+). Thus, the score is between 0 and 3. The optimal cutoff values of the IHC score were estimated using receiver operating characteristic (ROC) curve analysis. For correlation analysis, we defined score <0.5 and score ≥0.5 as low and high LINC00671, score <0.75, and score ≥0.75 as low and high STAT3 and LDHA, respectively.

### Statistical analysis

SPSS (version 23.0) or Prism GraphPad (version 8.0) was used for statistical analysis. All experiments in vitro were performed in triplicate and repeated three times. The statistical significance of cell proliferation, migration, and invasion assays and luciferase reporter gene assays were determined by a two-tailed Student’s *t* test. Spearman correlation analysis was performed using GraphPad Prism 8.0 to evaluate the relationship between different factors. The difference was considered statistically significant (*P* < 0.05).

## Results

### LINC00671 is a novel LDHA negatively-correlated lncRNA and associated with good clinical prognosis

**Fig. 1:**
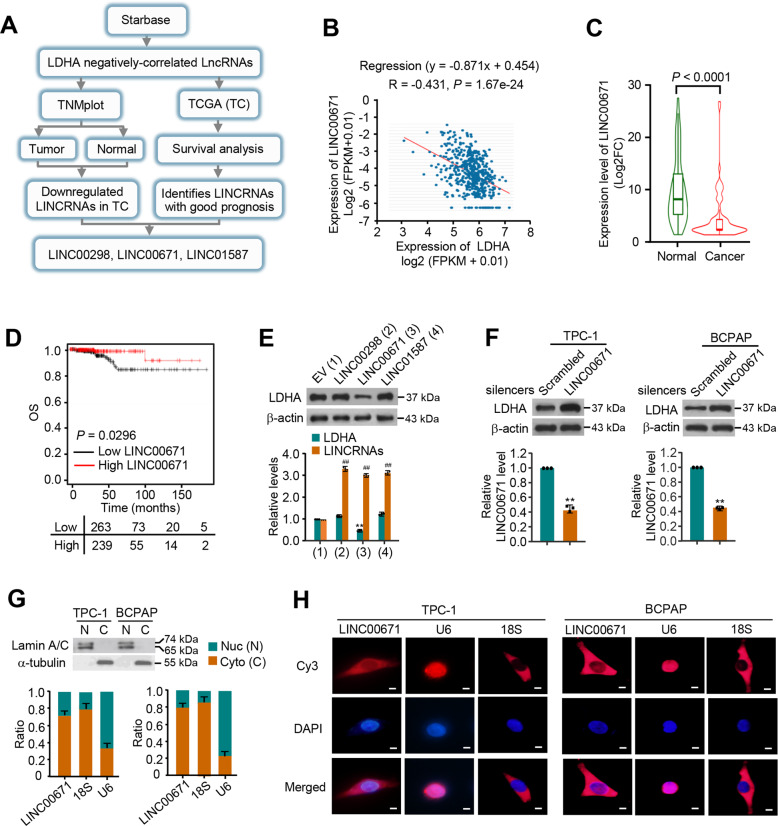
LINC00671 is a novel LDHA negatively-regulated LncRNA and correlated with good clinical prognosis. **A** Schematic of the screening strategy to identify lncRNAs that are downregulated in TC and negatively correlated with LDHA. **B** Pearson correlation analysis of the expression of LINC00671 and LDHA in TC tissue (http://starbase.sysu.edu.cn). **C** The expression of LINC00671 in TC patients and normal controls demonstrated by TNM plot under the item of compare tumor and normal section (https://www.tnmplot.com/). **D** Kaplan–Meier analysis of the overall survival rate (log-rank test, two sides) of TC patients with low (*n* = 361) or high (*n* = 310) expression of LINC00671 (http://kmplot.com/analysis/). **E** The protein levels and mRNA levels of LDHA in TPC-1 cells transfected with empty vector, LINC00298, LINC00671, and LINC01587 expression vectors. Orange bars show the levels of transfected LncRNAs including LINC00671. **F** The expression levels of LDHA and LINC00671 in TPC-1 cells (left panel) and BCPAP (right panel) transfected with scrambled and LINC00671 smart pool of silencers. The data are expressed as mean ± standard error. **G** The expression of LINC00671 in TPC-1 and BCPAP cells was detected by qRT-PCR. The separation of the nucleus and cytoplasm was estimated by western blot. Lamin A/C is used as a nuclear marker and tubulin is used as a cytoplasmic marker. **H** Subcellular localization of LINC00671 (red) in TPC-1 and BCPAP cells by combining RNA FISH and immunofluorescence. The nucleus was stained with DAPI (blue). Scale bar = 10 μm. All values shown are mean ± SD of triplicate measurements and have been repeated three times with similar results (**P < 0.01 versus corresponding control LDHA. ^##^*P* < 0.01 versus corresponding control of lncRNAs).

To screen out novel lncRNAs negatively correlated with LDHA, we analyzed the two databases of TC based on the screening strategy shown in Fig. 1A. In brief, LDHA negatively-correlated lncRNAs were firstly screened out from the Starbase database, the expression of lncRNAs between normal and cancerous tissues was compared, and then the survival of lncRNAs was analyzed based on TCGA database. Finally, we identified three lncRNAs negatively correlated with LDHA, LINC00671, LINC00298, and LINC01587, which demonstrated good clinical prognosis (Fig. 1B–D and Fig. S1A–F). To determine if the filtered three lncRNAs indeed downregulate the LDHA expression in cultured cells, we transfected two PTC cell lines TPC-1 and BCPAP with three lncRNAs, respectively. The results showed that only LINC00671 led to a marked decrease in both LDHA mRNA and protein levels in the two PTC cells (Fig. 1E and Fig. S1G). To further confirm the effect of LINC00671 in PTC cells, we knocked down LINC00671 with the specific smart pool of silencers and found that the LDHA expression levels were significantly increased in both TPC cells (Fig. 1F). These data indicate that LINC00671 downregulates the expression of LDHA in PTC cells. Since LINC00671 is a novel-identified PTC-related lncRNA, we next investigated the subcellular localization of LINC00671 in PTC cells by qRT-PCR analysis. The results demonstrated that LINC00671 mainly localizes in the cytoplasm, which was further confirmed by RNA fluorescence in situ hybridization analysis (FISH) (Fig. 1G, H).

### LINC00671 inhibits proliferation, migration, and invasion by downregulating the expression of LDHA in thyroid cancer cells

**Fig. 2:**
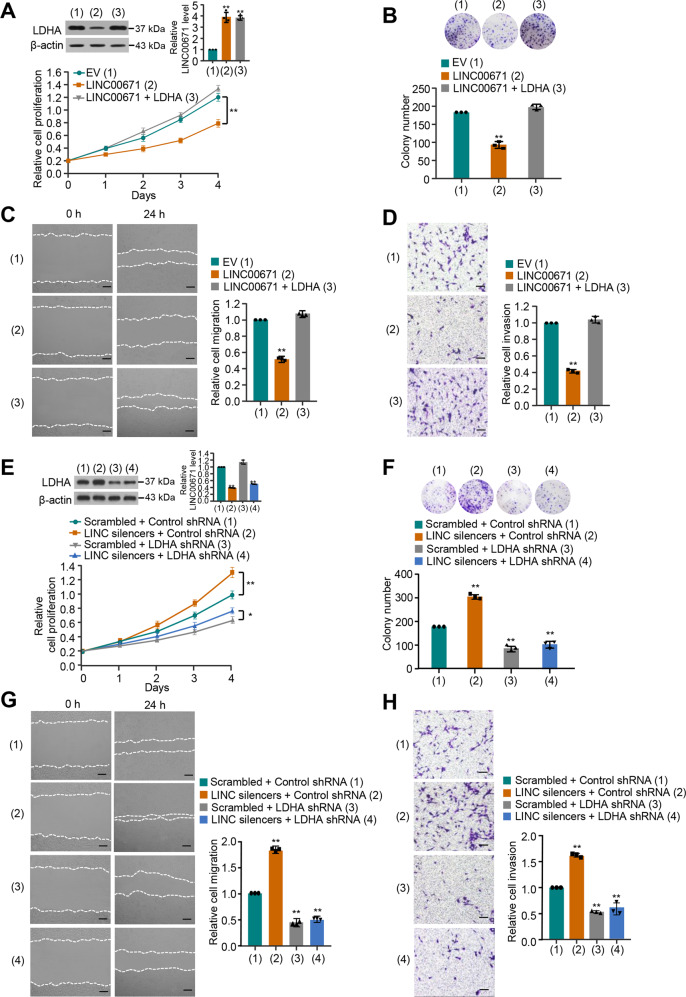
LINC00671 inhibits proliferation, migration, and invasion by downregulating the expression of LDHA in thyroid cancer cells. **A** TPC-1 cells were transfected with LINC00671 or LINC00671 plus LDHA expression vector. The cell proliferation was determined by the CCK-8 assay. The representative immunoblot shows the LDHA expression. The histogram shows the expression of LINC00671 as detected by qRT-PCR. **B** Colony formation assay of TPC-1 cells transfected as in (**A**). Representative image shows the colonies in plates (upper panels). The histogram shows colony number. **C**, **D** Wound healing (**C**) and Transwell assays (**D**) of TPC-1 cells transfected as in (**A**). Right histograms show relative cell migration and invasion. **E**, **F** Lentivirus-mediated LDHA knockdown (LDHA shRNA) or control TPC-1 cells were transfected with Scramble or LINC00671 smart pool of silencers and analyzed as in (**A**) and (**B**). **G**, **H** Wound healing (**G**) and Transwell (**H**) assays of lentivirus-mediated LDHA knockdown (LDHA shRNA) or control TPC-1 cells were transfected as in (**E** and **F**). Scale bar, 50 μm. All values shown are mean ± SD of triplicate measurements and have been repeated three times with similar results (*P < 0.05, **P < 0.01 versus corresponding control).

To study the biological function of LINC00671 in PTC cells, PTC cells with LINC00671 overexpression were analyzed for the cell growth, migration, and invasion. Cell proliferation and colony formation assays demonstrated that overexpression of LINC00671 suppressed the proliferation of TPC-1 and BCPAP cells. These effects could be reversed by LDHA reexpression in the LINC00671 transfected cell lines (Fig. 2A, B and Fig. S2A–B). In addition, overexpression of LINC00671 resulted in a decline in migration and invasion, which could be reversed by the reexpression of LDHA in the LINC00671 transfected cells (Fig. 2C, D and Fig. S2C–D). Meanwhile, LDHA knockdown abolished the ability of LINC00671 to regulate the proliferation, migration, and invasion of PTC cells, indicating that LINC00671 suppresses PTC cell proliferation, migration, and invasion through inhibition of LDHA expression (Fig. 2E–H and Fig. S2E–H).

### LINC00671 suppresses glycolysis by downregulating the expression of LDHA in thyroid cancer cells

**Fig. 3:**
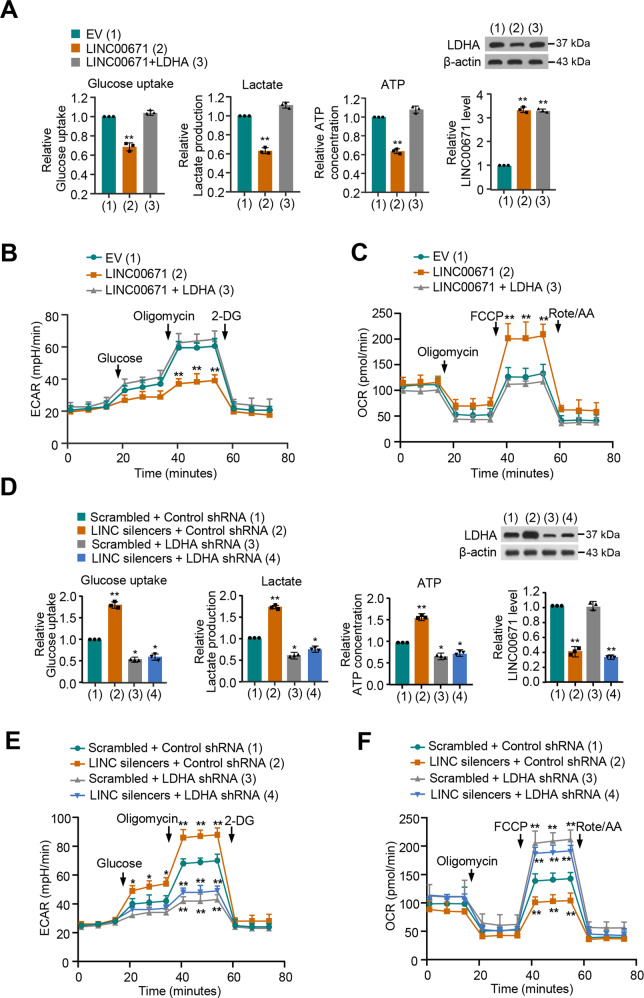
LINC00671 suppresses glycolysis by downregulating the expression of LDHA in thyroid cancer cells. **A** TPC-1 cells were transfected with LINC00671 or LINC00671 plus LDHA expression vector. Glucose uptake and the production of lactate and ATP were determined. Representative immunoblot reveals the expression of LDHA. qRT-PCR analysis indicates the LINC00671 expression. **B**, **C** TPC-1 cells were transfected as in (**A**), and extracellular acidification rate (ECAR) (**B**) and oxygen consumption rate (OCR) (**C**) were then evaluated. LDHA shRNA or control TPC-1 cells were transfected with Scramble or LINC00671 smart pool of silencers. **D** Glucose uptake, lactate production, and ATP production were measured. Typical immunoblot reveals the expression of LDHA. qRT-PCR analysis shows the LINC00671 expression. **E**, **F** ECAR (**E**) and OCR (**F**) assays of LDHA knockdown or control TPC-1 cells were transfected as in (**D**). All values shown are mean ± S.D. of triplicate measurements and have been repeated three times with similar results (**A**–**F**). *P < 0.05 versus corresponding NC. **P < 0.01 versus corresponding NC.

LDHA promotes the glycolysis process by catalyzing the conversion of pyruvate to lactate acid [14]. Therefore, we investigated whether LINC00671 affects glycolysis through LDHA in PTC cells. We initially tested the regulation of LINC00671 on glucose uptake, lactate production, and ATP production. As expected, LINC00671 inhibited glucose uptake, lactate production, and ATP production. These effects could be reversed by LDHA reexpression in the LINC00671-transfected cells (Fig. 3A and Fig. S3A). LINC00671 also exhibited a reduced extracellular acidification rate (ECAR), which reflects the total glycolytic flux, and increased oxygen consumption rate (OCR), which is an indicator of mitochondrial respiration (Fig. 3B, C and Fig. S3B–C). Likewise, reexpression of LDHA in PTC cells with LINC00671 overexpression restored these effects. In addition, LDHA knockdown abolished the ability of LINC00671 to regulate glucose uptake, lactate production, and ATP production of PTC cells, indicating that LINC00671 suppresses PTC cells glucose uptake, lactate production, and ATP production through inhibition of LDHA expression (Fig. 3D–F and Fig. S3D–F). Taken together, these data collectively suggest that LINC00671 suppresses glycolysis via inhibition of LDHA expression in PTC cells.

### Hypoxia suppresses LINC00671 expression and activates LDHA expression largely through regulating STAT3 transcription

**Fig. 4:**
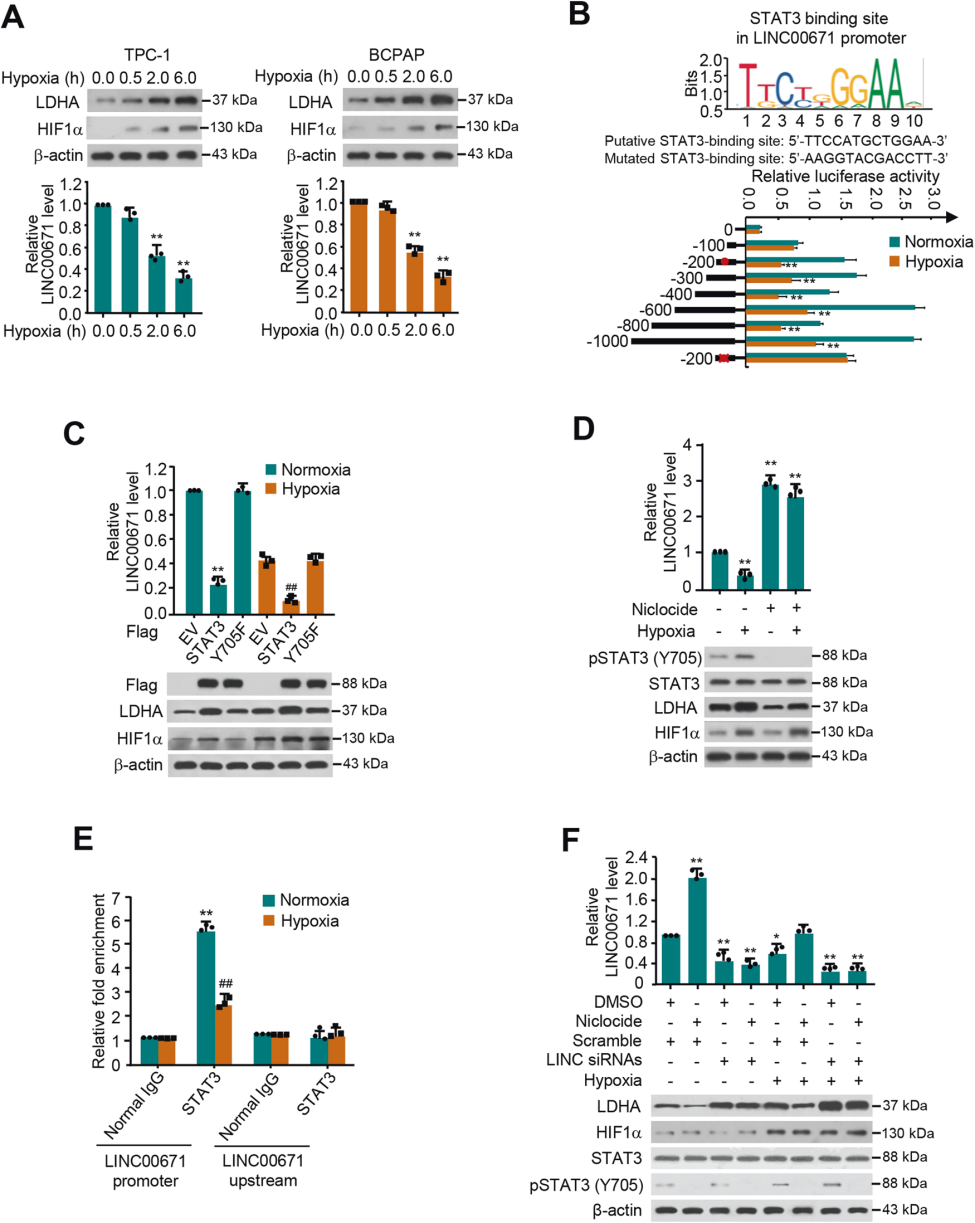
Hypoxia inhibits LINC00671 expression and activates LDHA expression largely through STAT3 transcription. **A** qRT-PCR and Western blot analysis of TPC-1 and BCPAP cells exposed to normoxia (0 h) or hypoxia at different times (0.5, 2, 6 h). The representative immunoblot shows the expression of LDHA and HIF-1α. β-actin was used as a loading control. The expression level of LINC00671 was determined by qRT-PCR. **B** JASPAR (http://jaspar.genereg.net/) predicts that the STAT3 binding element on the LINC00671 promoter is conserved. TPC-1 cells transfected with different LINC00671 constructs or empty vector and exposed to normoxia or hypoxia, the luciferase activity of the different LINC00671 promoter-reporter genes was measured. The solid circle shows the position of the putative STAT3 binding site, and the “X” shows the mutated STAT3 binding site. The red letters in each binding region indicate a putative STAT3 binding sequence or a mutated STAT3 binding sequence. **C** qRT-PCR analysis of LINC0071 expression in TPC-1 cells transfected with EV, STAT3, and STAT3 (Y705F), and exposed to normoxia or hypoxia condition. A representative immunoblot shows the expression of LDHA and HIF-1α. β-actin was used as an internal control. **D** qRT-PCR analysis of LINC00671 expression in TPC-1 cells treated with Niclocide with or without hypoxia. **E** ChIP analysis of LINC00671 promoter or STAT3 occupancy rate upstream of the promoter in TPC-1 cells under normoxia or hypoxia. **F** qRT-PCR analysis of LINC00671 expression in TPC-1 cells transfected with LINC00671 siRNAs, treated with Niclocide, and exposed to hypoxia or not. The representative immunoblot shows the expression of LDHA, HIF-1α, and pSTAT3 (Y705). β-actin was used as a loading control. The displayed values are the mean ± standard deviation. The triplicate measurement results were repeated 3 times and the results were similar. *P < 0.05, **P < 0.01, relative to the corresponding empty vector. ^##^*P* < 0.01 versus the corresponding empty carriers under hypoxic conditions.

Hypoxic microenvironment is a common feature of tumor [15], therefore, we investigated if hypoxia affects LINC00671/LDHA axis. Intriguingly, hypoxia not only stimulated the LDHA expression, but decreased that of LINC00671 expression as well (Fig. 4A). To further determine how hypoxia represses LINC00671 expression in PTC cells, we investigated the response of LINC00671 promoter after hypoxia. Analysis of various LINC00671 promoter deletion reporter constructs demonstrated that the promoter region from −300 to −200 bp contained a hypoxia-repressive element (Fig. 4B). Mutation of a putative STAT3-binding site in this region predicted by a bioinformatics method (http://tfbind.hgc.jp) resulted in loss of the repression.

STAT3 is a key transcription factor involved in a variety of growth factors and cytokines, which triggers a variety of biological processes, including cell growth, differentiation, and survival [16]. Niclocide has been proven to be an effective inhibitor of STAT3, which disrupts the transcriptional activity of STAT3 by inhibiting the phosphorylation of tyrosine 705 residue (Y705) and the nuclear translocation of STAT3 [17, 18]. As expected, either under normoxia or hypoxia condition, STAT3 instead of STAT3 functional mutant (Y705F) affects LINC00671 and LDHA expression. Besides, the STAT3 inhibitor, Niclocide significantly increased the level of LINC00671, and more importantly, Niclocide greatly impaired the effect of hypoxia on LINC00671. These data indicate that STAT3 is the specific transcription factor for LINC00671 regulation and the site of Y705 is important for its regulation (Fig. 4C, D and Fig. S4A). Chromatin immunoprecipitation (ChIP) assay further displayed that, under normoxia, STAT3 was recruited to the region containing the putative STAT3-binding site within the LINC00671 promoter, but not to a region upstream of the LINC00671 promoter, and the recruitment was decreased under hypoxia (Fig. 4E). Niclocide increased the LINC00671 level and decreased the LDHA level. Importantly, Niclocide greatly impaired the ability of hypoxia to regulate LINC00671 and LDHA expression (Fig. 4F and Fig. S4B). In addition, knockdown of LINC00671 greatly impaired the effect of hypoxia on LDHA upregulation. These data suggest that hypoxia inhibits LINC00671 expression and activates LDHA expression largely through STAT3 transcription.

### STAT3 increases proliferation, migration, and invasion of thyroid cancer cells and activates glycolysis via regulation of LINC00671 expression

**Fig. 5:**
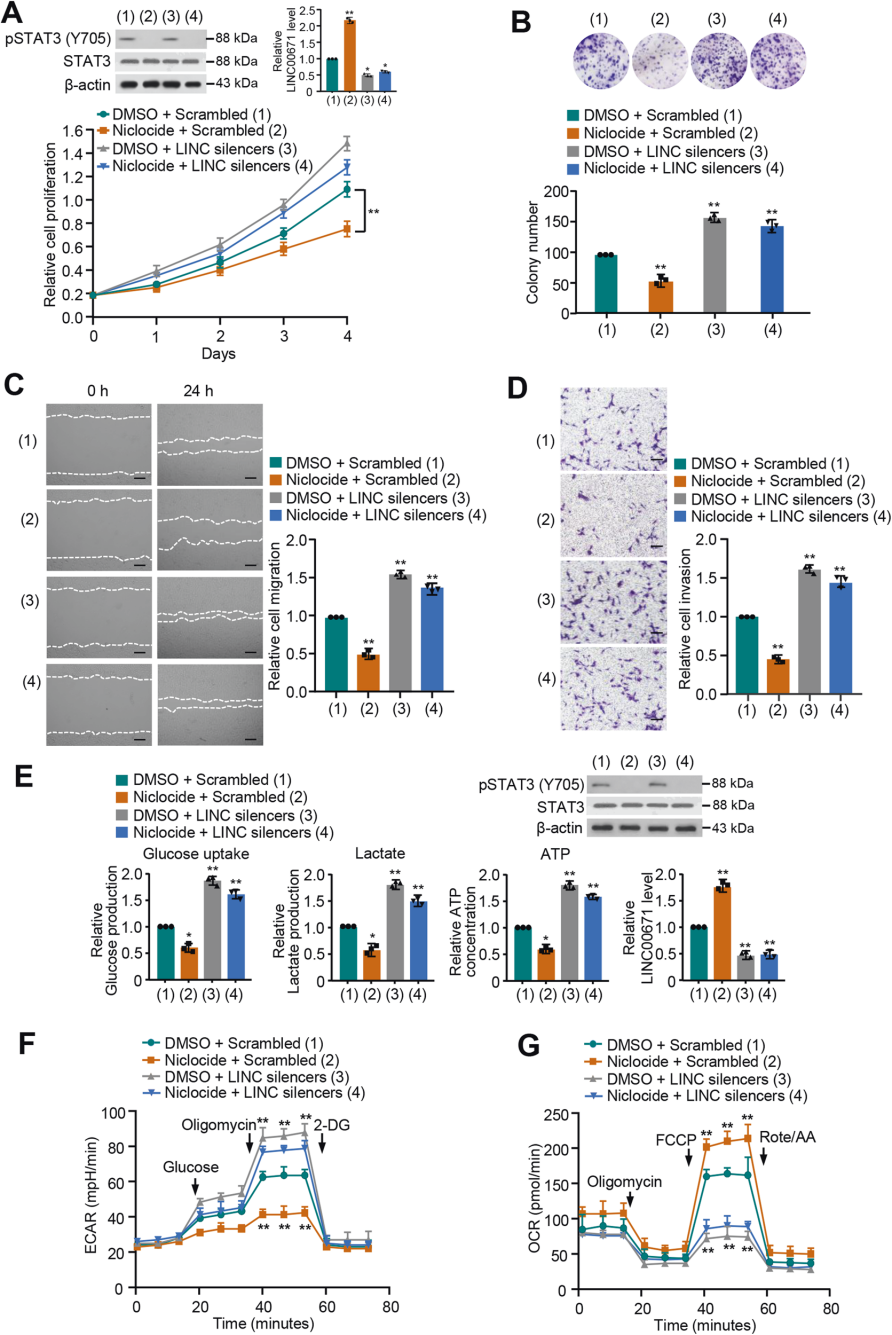
STAT3 increases proliferation, migration, and invasion of thyroid cancer cells and activates glycolysis via regulation of LINC00671 expression. **A** TPC-1 cells were transfected with LINC00671 smart pool of silencers and treated with Niclocide. The proliferation of the cells was detected by CCK-8 assay. The representative immunoblot shows pSTAT3 level. Histograms show LINC00671 expression determined by qRT-PCR. **B** Colony formation assay of TPC-1 cells transfected and treated as in (**A**). Representative images show colonies in plates (upper panels). Histograms show colony number. **C**, **D** Wound healing (**C**) and Transwell (**D**) assays of TPC-1 cells transfected and treated as in (**A**). Right histograms show the relative cell migration and invasion. **E** Glucose uptake and the production of lactate and ATP were determined. Representative immunoblot reveals the expression of pSTAT3. qRT-PCR analysis indicates LINC00671 expression. **F**, **G** TPC-1 cells were transfected and treated as in (**A**), and extracellular acidification rate (ECAR) (**F**), and oxygen consumption rate (OCR) (**G**) were then measured. The displayed values are the mean ± standard deviation. The triplicate measurement results were repeated 3 times and the results were similar. *P < 0.05, **P < 0.01 versus the corresponding control. Scale bar, 50 μm.

To investigate whether STAT3 regulates these effects through LINC00671, we knocked down LINC00671 in PTC cells and treated with Niclocide. Cell proliferation and colony formation assays revealed that Niclocide suppressed PTC cell growth, and LINC00671 knockdown increased that of PTC cell growth (Fig. 5A, B and Fig. S5A). Importantly, LINC00671 knockdown greatly attenuated the ability of Niclocide to regulate PTC cell proliferation, suggesting that STAT3 increases PTC cell proliferation dependent on the regulation of the LINC00671 expression.

**Fig. 6:**
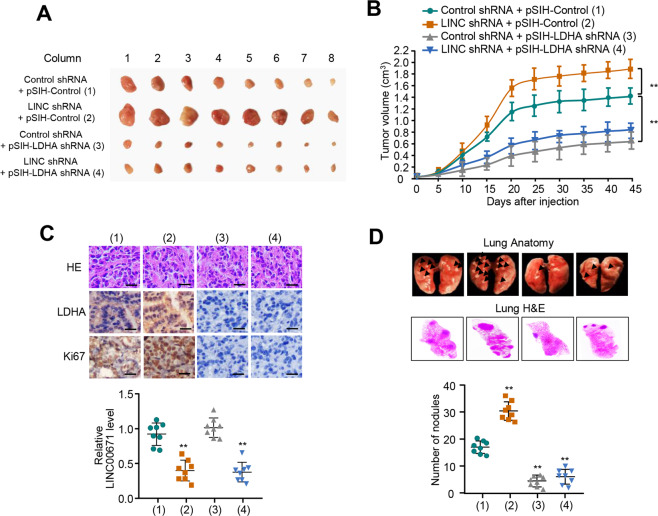
LINC00671/LDHA axis regulates TC tumor growth and lung metastasis in vivo. **A**, **B** TPC-1 cells stably infected with the lentivirus carrying the indicated constructs were injected subcutaneously into nude mice (*n* = 8 per group). The tumor volume was measured every 5 days and the growth curve was plotted (**B**). **C** Representative IHC staining of LDHA, Ki67, and H&E images of tumors resected from (**A**). Scale bar, 50 µm. The scattered plot shows the expression levels of LINC00671 measured by qRT-PCR. **D** TPC-1 cells stably expressing the constructs were injected through the tail vein to establish a thyroid cancer cell metastasis model in nude mice (*n* = 8 per group). Anatomical and histological analyses of representative lung metastases were performed. The number of tumor nodules was observed under the anatomical microscope. Symbols represent individual mice. **P < 0.01 versus the corresponding control.

Next, we determined whether STAT3 increases PTC cell migration, invasion, and glycolysis via LINC00671. As expected, Niclocide reduced the PTC cell migration and invasion (Fig. 6C, D and Fig. S5B–C). LINC00671 knockdown greatly attenuated the ability of Niclocide to suppress PTC cell migration and invasion. In addition, Niclocide lowered the levels of glucose uptake, lactate production, and ATP generation, and displayed decreased ECAR and increased OCR. Again, LINC00671 knockdown greatly impaired the ability of Niclocide to regulate PTC cell migration, invasion, and glycolysis (Fig. 6C–G and Fig. S5D–F). Similar phenotypes could be observed in STAT3 knockdown cells (Fig. S6). Taken together, these results suggest that STAT3 increases proliferation, migration, and invasion of thyroid cancer cells and activates glycolysis via regulation of LINC00671 expression.

### LINC00671/LDHA axis regulates PTC tumor growth and lung metastasis in vivo

In order to determine the in vivo phenotype of the LINC00671/LDHA axis, we examined the effect of the axis on TC growth by injecting TPC cells harboring the indicated constructs into the back of BALB/c nude mice. As expected, LINC00671 knockdown dramatically enhanced TC tumor growth. In contrast, tumor growth was inhibited when LDHA was knocked down. More importantly, the effect of LINC00671 knockdown on tumor growth was markedly abrogated when LDHA was knocked down (Fig. 6A–C).

As metastasis occurs in ~10% of patients with thyroid cancer, and about a half of the distant metastasis occurs in lung [19, 20]. We investigated the effect of the pathway on PTC tumor metastasis. The number of the nodules spread throughout the pulmonary region was markedly increased in LINC00671 knockdown group compared with that in control group (Fig. 6D). On the contrary, LDHA knockdown resulted in decreased metastatic spread of PTC cells to the lung. Importantly, LDHA knockdown greatly impaired the ability of LINC00671 knockdown to regulate lung metastasis (Fig. 6D). Histologic analysis on the lungs confirmed the metastasis foci. Taken together, these data suggest that LINC00671/LDHA axis regulates PTC tumor growth and lung metastasis in vivo.

### Correlation between LINC00671, LDHA, and STAT3 expression and association of LINC00671 with glucose uptake in patients with thyroid cancer

**Fig. 7:**
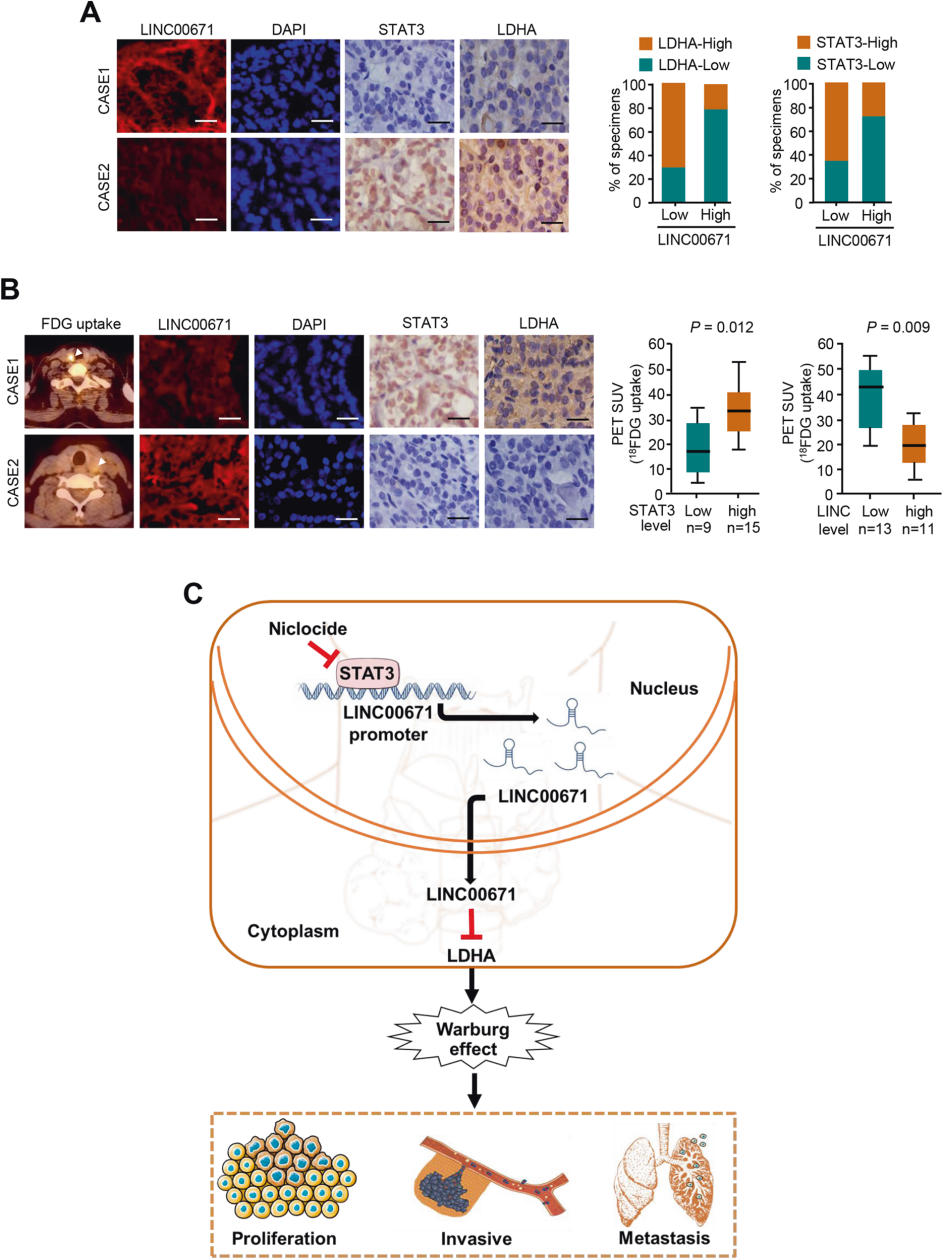
Correlation between the expression of LINC00671 and LDHA and association of LINC00671 with glucose uptake in patients with thyroid cancer. **A** Representative IHC staining for STAT3, LDHA, and FISH staining for LINC00671 in PTC patients. Scale bar, 50 μm. Right panel showing the percentage of specimens with low or high LDHA and STAT3 expressions in the low or high LINC00671 expression groups. CASE 1 and CASE 2 refer to two representative samples categorized by low and high LINC00671 expression. **B** The correlation of glucose uptake in thyroid cancer patients with different expressions of LDHA, STAT3, and LINC00671 using the Mann–Whitney *U* test. CASE 1 and CASE 2 refer to two representative samples categorized by low and high FDG uptake. Scale bar, 50 μm. **C** Graphical abstract underlying the role of the STAT3/LINC00671/LDHA axis in regulating PTC growth and lung metastasis.

We assessed LDHA expression by immunohistochemical staining (IHC) and LINC00671 expression by FISH in 45 human PTC samples. In consistent with the investigations that LINC00671 inhibits LDHA expression and STAT3 inhibits LINC00671 expression in cultured cells, the expression of LINC00671 was negatively correlated with LDHA and STAT3 expression. Moreover, the expression of STAT3 positively correlated with LDHA expression (Fig. 7A). Importantly, patients with PTC cancers who had increased glucose uptake assessed by ^18^FDG PET scans displayed lower levels LINC00671 and higher levels of STAT3 and LDHA expression (Fig. 7B). The specificity of LINC00671 was confirmed by FISH and the specificity of the LDHA and STAT3 antibody was confirmed by IHC and immunoblotting with cell lysates (Fig. S7). Taken together, these data suggest the important pathological role of the STAT3/LINC00671/LDHA axis in PTC.

## Discussion

The common feature of cancer cell is the altered metabolism by the increased glucose uptake and fermentation of glucose to lactate, the phenomenon of which is known as the aerobic glycolysis (also known as “Warburg Effect”) [21]. This metabolic rewiring brings cancer cell a growth advantage to promote survival, proliferation, and long-term maintenance. Thus, targeting the process of aerobic glycolysis has been recognized to be an effective way in governing the tumor growth and progression as well as enhancing the effect of anticancer treatments [22]. In the glycolytic pathway, enzymes have drawn the most attentions as chosen for the targets of cancer treatment, motivating researchers to search for new agents targeting the metabolic enzymes that block glycolysis or induce a switch from glycolysis to mitochondrial respiration.

LDHA, as one of the key glycolytic enzyme, catalyzes the reduction of pyruvate to lactate acid by compensating for the decreased function of oxidized mitochondria and maintains cell survival under hypoxic conditions [23, 24]. The aberrant expression and upregulation of LDHA are closely related to a variety of cancers and associated with poor prognosis of patients with cancers, including thyroid cancer [25, 26]. LDHA increases proliferation, invasion, and metastasis of thyroid cancer cells and helps thyroid cancer cells escape from immunity [27]. Therefore, LDHA is considered as a promising target for thyroid cancer prevention and treatment. Chemical inhibitors targeting LDHA are being developed [28]. However, the regulatory mechanisms of LDHA inhibition and the physiological significance of LDHA inhibitors in PTC are still unknown. Our research identified a novel STAT3/LINC00671/LDHA axis to regulate glycolysis, growth, and metastasis of thyroid cancer. STAT3 and LINC00671 are novel upstream regulators of LDHA. In PTC cells, LINC00671 inhibits glucose uptake and lactate production, and induces a switch from glycolysis to mitochondrial respiration through inhibition of LDHA expression.

LINC00671 may inhibit glucose uptake by inhibiting LDHA. LINC00671 suppresses PTC tumor growth and metastasis through inhibition of LDHA-mediated Warburg effect. We further showed that LINC00671 is a new transcriptional target of STAT3. Moreover, in thyroid cancer samples, STAT3 or LINC00671 expression is negatively correlated with LDHA expression as well as increased tumor FDG uptake. Thus, our data establish the physiological and pathological significance of STAT3 or LINC00671 in regulating LDHA-mediated Warburg effect. LINC00671 or STAT3 activation may be useful for treatment of thyroid cancer with LDHA overexpression.

With the continuous update and development of bioinformatics technology, a large amount of long non-coding RNA (lncRNA) has been identified. Non-coding RNA (ncRNA) is a new class of transcripts, although untranslated, plays a vital role in varieties of cellular and physiological activities, such as chromatin imprinting, cell differentiation, and tumorigenesis, etc. [29, 30]. As for regulation of tumorigenesis and progression, several lncRNAs are reported to be abnormally expressed in multiple cancers, and regulate tumor cell proliferation, differentiation, apoptosis, migration, and invasion [11, 31]. Some lncRNAs have been reported to be involved in tumor glycolysis by regulating LDHA. gluclncRNA (HULC) is up-regulated in hepatocellular carcinoma (HCC), directly binds LDHA, enhances the binding of LDHA to fibroblast growth factor receptor type 1 (FGFR1), thus promoting the glycolysis, proliferation, and progression of HCC cells [32]. Yin et al. reported that LncRNA DUXAP8 promotes cell survival, migration, and glycolysis of non-small cell lung cancer by upregulation of LDHA via sponging of miR-409-3p [32]. Yan et al. reported that lncRNA IGFBP4-1 reprograms energy metabolism to promote lung cancer progression by affecting LDHA expression [33]. Tang et al. reported that LncRNA GLCC1 stabilizes the ubiquitination of c-Myc transcription factors and further increases the expression of c-Myc target genes (such as LDHA), thus reprogramming glucose metabolism to promote the proliferation of rectal cancer [34]. In thyroid cancer, several lncRNAs, such as MALAT1, H19, BANCR, HOTAIR have been identified as contributing factors to the development of cancer [12]. However, whether lncRNAs regulate the above-mentioned functions via controlling LDHA-mediated glycolysis remains unknown. To our knowledge, LINC00671 is the first lncRNA discovered to regulate LDHA-mediated glycolysis in thyroid cancer.

As is well-known, the main function of LDHA is catalyzing the conversion of pyruvate to lactate, which is the final step of glycolysis. However, some research groups also found that LDHA processes the ability to increase the glucose uptake [35, 36]. They found the decreased glucose uptake brought by LDHA inhibition was not due to the reduced cell density or reduced surface expression of GLUT1 [36]. In deeper exploration of the mechanism of how LDHA inhibition affects the glucose uptake, they found that LDHA inhibition suppresses NAD^+^ regeneration and compromises the activity of glyceraldehyde 3‐phosphate dehydrogenase (GAPDH), an enzyme required for the conversion of glyceraldehyde 3‐phosphate to 1,3‐biphosphoglycerate [37]. This in turn triggers a build‐up of the glycolytic intermediates in the first few steps of glycolysis, increasing in cellular levels of unused glucose and the suppression in glucose uptake [37]. Based on the previous findings that LDHA regulates the glucose uptake, we speculated that LINC00671 regulates glucose uptake via downregulation of LDHA expression. Whether there are other mechanisms for LINC00671 to regulate glucose uptake still need investigation.

Researchers have been engaged in the mechanisms underlying the JAK2/STAT3 pathway mediating tumorigenesis for years [38, 39]. Once JAK2 protein was activated, STAT3 can be phosphorylated at Tyr-705, formed dimerization, and translocated to the cell nucleus, in which p-STAT3 functions as transcription factor relying on its DNA-binding domain to regulate downstream genes expression. STAT3 signaling is critical for driving tumor growth, migration, angiogenesis, and inflammatory cross-talk with immune cells during the carcinogenesis [40, 41]. Studies have shown that the expression of STAT3 and pSTAT3 in PTC tissues is significantly higher than that of adjacent tissues [42]. In addition, the expression of STAT3 and pSTAT3 in the group with lymph node metastasis is significantly higher than that in the group without lymph node metastasis [43]. However, the inhibitor of the STAT3 pathway during the development of PTC is still obscure. Niclocide (C_13_H_8_C_l2_N_2_O_4_, MW: 327.117), a drug anthelmintic approved by the Food and Drug Administration (FDA), has proven to be an effective inhibitor of STAT3 in a concentration- and time-dependent manner [44]. Niclocide disrupts the transcriptional activity of STAT3 by inhibiting the phosphorylation of Tyr-705 site and the nuclear translocation of STAT3 [17, 44]. Niclocide has been reported to inhibit the growth of various cancer cells, such as breast cancer, lung cancer, head and neck cancer, and melanoma [45, 46]. In this study, we observed both in vitro and in vivo that the Niclocide not only suppresses thyroid cancer cell proliferation, migration, and invasion, but suppresses glycolytic abilities of thyroid cancer cell as well. Herein, we provide the first evidence that Niclocide is an inhibitor of tumor growth and metastasis in PTC, complementing its biological function via influencing the dynamic properties of regulating JAK2/STAT3 signaling network. Utilization of Niclocide will be a promising treatment for combating PTC growth and metastasis.

## Supplementary information

Figure S1-S12

Table S1 & Table S2

Declaration of contributions to article

Checklist form

Pre-Authorship form

## Data Availability

All data generated or analyzed during this study are included in this published article and its supplementary information files.
