# Supplementary material for: STAT3/LINC00671 axis regulates papillary thyroid tumor growth and metastasis via LDHA-mediated glycolysis
Source: Cell Death Dis. 2021 Aug 17;12(9):799. doi: 10.1038/s41419-021-04081-0 (PMC8371129; doi:10.1038/s41419-021-04081-0)
Supplement: Supplementary file 1 — Figure S1-S12 [file 41419_2021_4081_MOESM1_ESM.pdf]

Figure S1

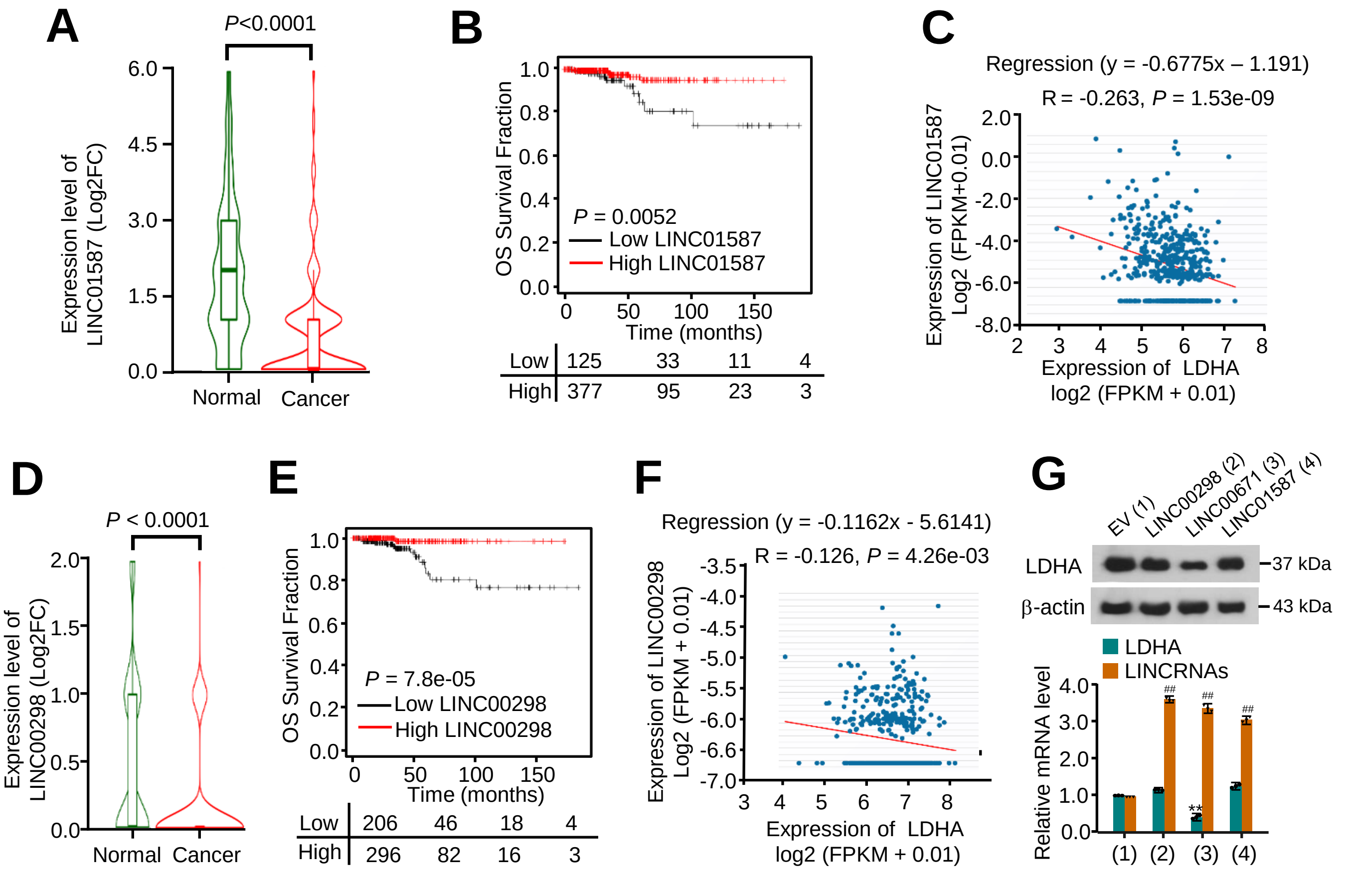

Supplementary Figure 1. Identification of LINC00671 as a lncRNA that is downregulated in TC and negatively correlated with LDHA. (A) The TNMplot revealed the expression of LINC01587 in TC patients and normal controls (P <0.0001). (B) Kaplan-Meier analysis (log-rank test, both sides) of the overall survival of TC patients with low (n = 173) or high (n = 498) LINC01587 expression (http://kmplot.com/analysis/). (C) Pearson's correlation analysis of LINC01587 and LDHA expression in TC tissue (http://starbase.sysu.edu.cn/). (D) The expression of LINC00298 in TC patients and normal controls revealed by the TNMplot (P <0.0001). (E) Kaplan-Meier analysis (log-rank test, two sides) of the overall survival of TC patients with low expression (n = 274) or high expression (n = 397) of LINC00298 (http://kmplot.com/ analysis/). (F) Pearson's correlation analysis of the expression of LINC00298 and LDHA in TC patients (http://starbase.sysu.edu.cn/). (G) The protein and mRNA levels of LDHA in BCPAP cells transfected with the empty vector, LINC00298, LINC00671 and LINC01587 expression vectors. Orange bars show the levels of transfected LINCRNAs including LINC00671 All values shown are mean ± SD of triplicate measurements and have been repeated 3 times with similar results. **P < 0.01 versus corresponding control LDHA. ##P < 0.01 versus corresponding control of LINCRNAs.

Figure S2

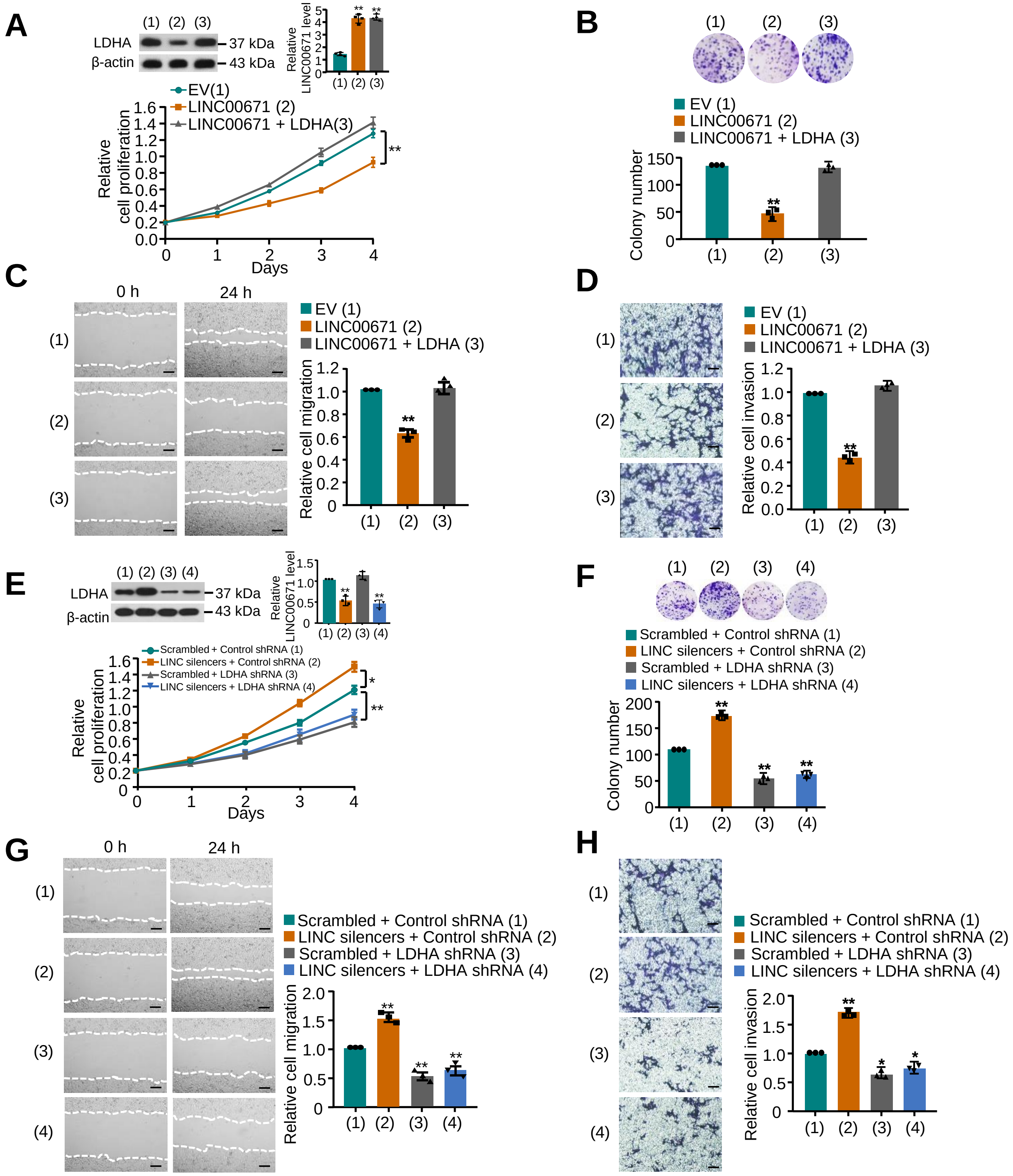

Supplementary Figure 2. LINC00671 inhibits proliferation, migration and invasion by inhibiting the expression of LDHA in Thyroid cancer cells. (A) BCPAP cells were transfected with LINC00671 or LINC00671 plus LDHA expression vector. The cell proliferation was detected by CCK-8 assay. The representative immunoblot shows LDHA expression. The histogram shows the expression of LINC00671 determined by qRT-PCR. (B) Colony formation assay of BCPAP cells transfected as in (A). Representative image shows the colonies in plates (upper panels). The histogram shows colony number. (C and D) Wound healing (C) and Transwell assays (D) of BCPAP cells transfected as in (A). Right histograms show relative cell migration and invasion. (E and F) Lentivirus-mediated LDHA knockdown (LDHA shRNA) or control BCPAP cells were transfected with Scramble or LINC00671 smart pool of silencers and analyzed as in (A) and (B). (G and H) Wound healing (G) and Transwell (H) assays of lentivirus-mediated LDHA knockdown (LDHA shRNA) or control BCPAP cells were transfected as in (E and F). All values shown are mean ± SD of triplicate measurements and have been repeated 3 times with similar results. *$P < 0.05$, **$P < 0.01$ versus corresponding control. Scale bar, 50 μm.

Figure S3

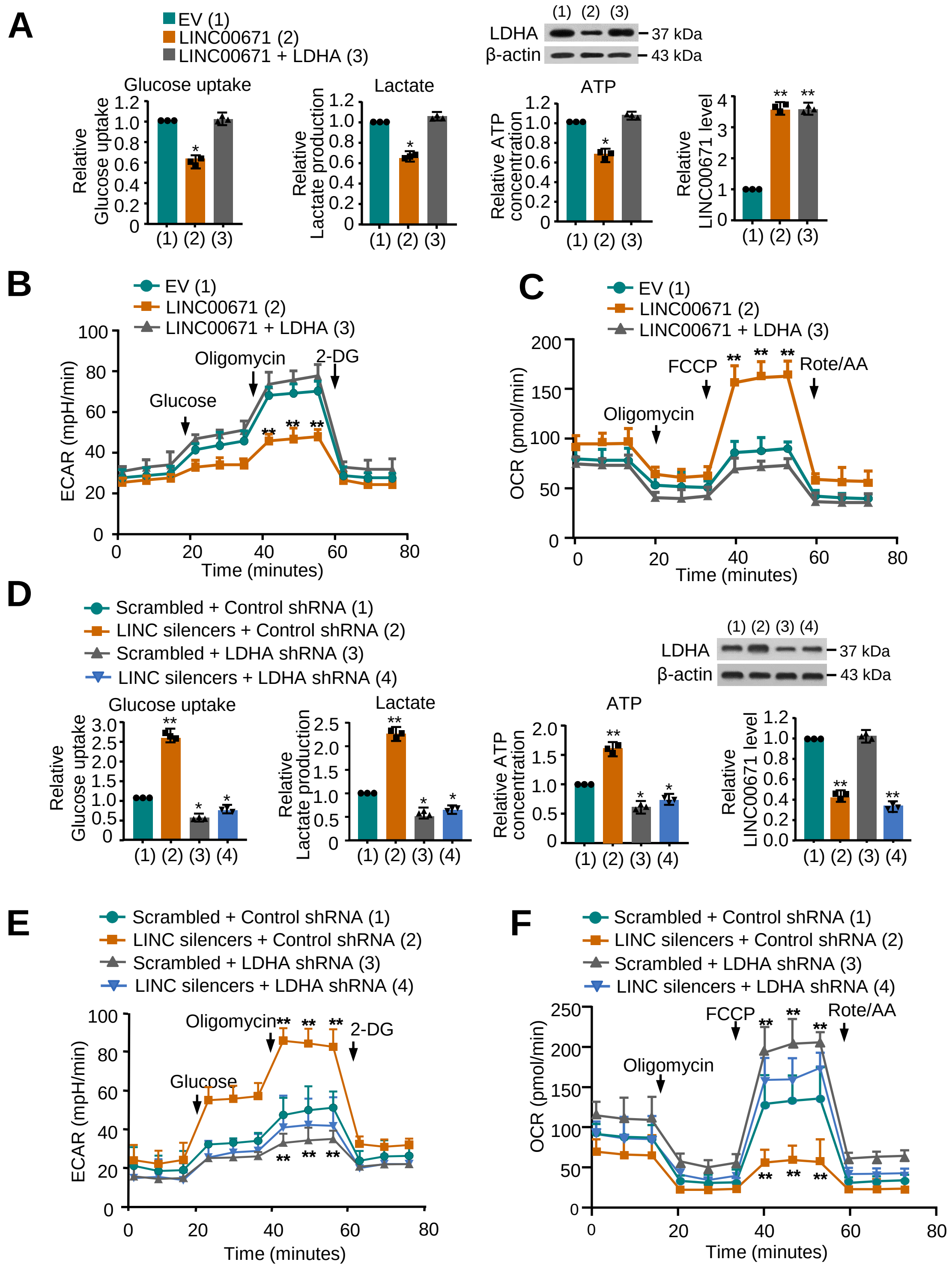

Supplementary Figure 3. LINC00671 suppresses glycolysis by downregulating the expression of LDHA in thyroid cancer cells. (A) BCPAP cells were transfected with LINC00671 or LINC00671 plus LDHA expression vector. Glucose uptake and the production of lactate and ATP were determined. Representative immunoblot reveals the expression of LDHA. qRT-PCR analysis indicates the LINC00671 expression. (B and C) BCPAP cells were transfected as in (A), and extracellular acidification rate (ECAR) (B) and oxygen consumption rate (OCR) (C) were then evaluated. LDHA shRNA or control BCPAP cells were transfected with Scramble or LINC00671 smart pool of silencers. (D) Glucose uptake, lactate production and ATP production were measured. Typical immunoblot reveals the expression of LDHA. qRT-PCR analysis shows the LINC00671 expression. (E and F) ECAR (E) and OCR (F) assays of LDHA knockdown or control BCPAP cells were transfected as in (D). All values shown are mean ± S.D. of triplicate measurements and have been repeated 3 times with similar results (A-F). *P < 0.05, **P < 0.01 versus the corresponding control.

Figure S4

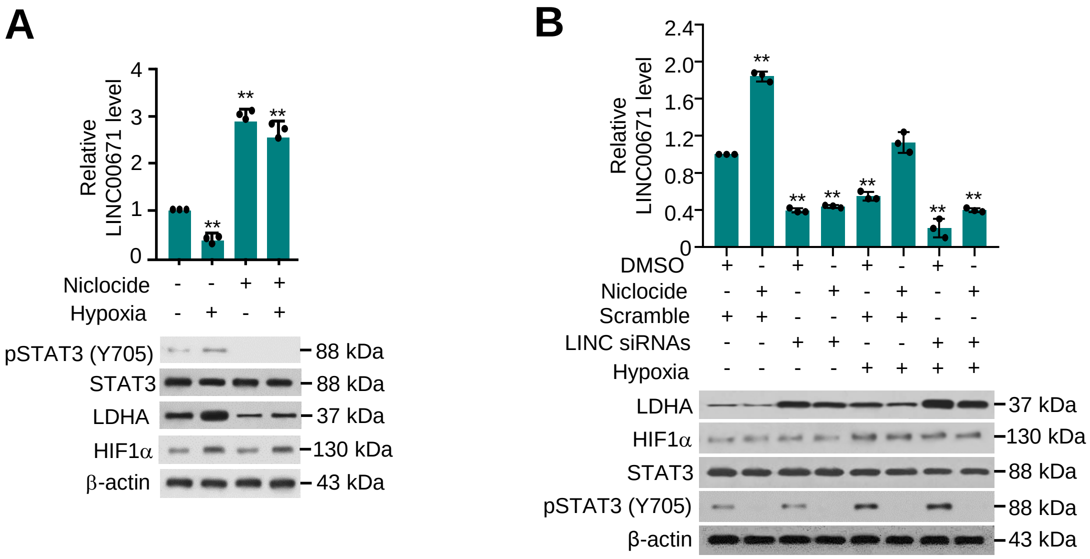

Supplementary Figure 4. Hypoxia inhibits LINC00671 expression and activates LDHA expression largely through STAT3 transcription. (A) qRT-PCR analysis of LINC00671 expression in BCPAP cells treated with Niclocide with or without hypoxia. (B) qRT-PCR analysis of LINC00671 expression in BCPAP cells transfected with LINC00671 siRNAs, treated with Niclocide and exposed to hypoxia or not. The representative immunoblot shows the expression of LDHA, HIF-1α and pSTAT3 (Y705). β-actin was used as a loading control. The displayed values are the mean ± standard deviation. The triplicate measurement results were repeated 3 times and the results were similar. ***P* < 0.01 relative to the corresponding empty vector.

Figure S5

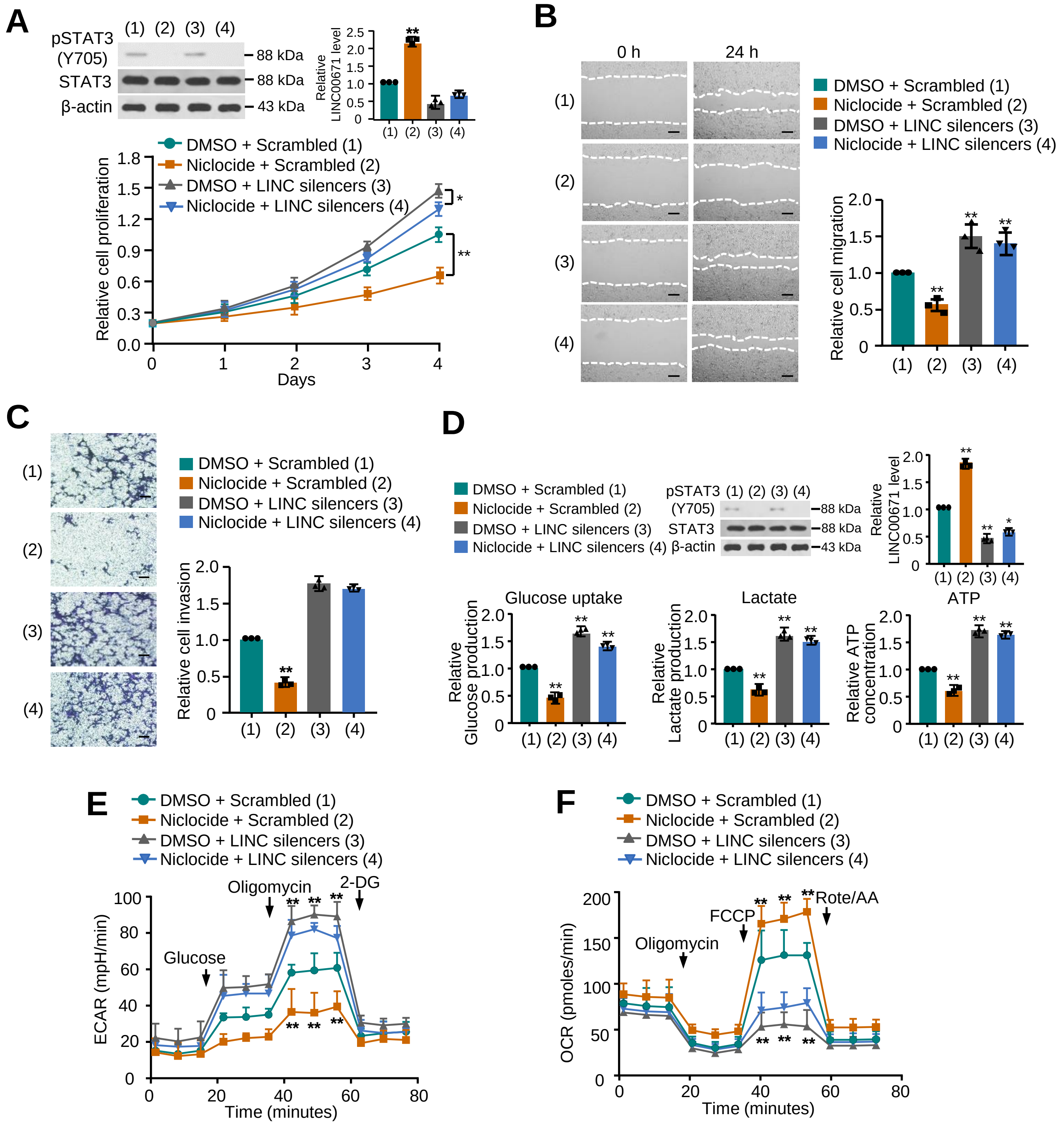

Supplementary Figure 5. STAT3 increases proliferation, migration and invasion of thyroid cancer cells and activates glycolysis via regulation of LINC00671 expression. (A) BCPAP cells were transfected with LINC00671 smart pool of silencers and treated with Niclocide. The proliferation of the cells was detected by CCK-8 assay. The representative immunoblot shows pSTAT3 level. Histograms show LINC00671 expression determined by qRT-PCR. (B and C) Wound healing (B) and Transwell (C) assays of BCPAP cells transfected and treated as in (A). Right histograms show the relative cell migration and invasion. (D) Glucose uptake and the production of lactate and ATP were determined. Representative immunoblot reveals the expression of pSTAT3. qRT-PCR analysis indicates LINC00671 expression. (E and F) BCPAP cells were transfected and treated as in (A), and extracellular acidification rate (ECAR) (E) and oxygen consumption rate (OCR) (F) were then measured. The data shown is the average ± SD of three measurements, which has been repeated 3 times and the results are similar. *P < 0.05, **P < 0.01 versus the corresponding control. Scale bar, 50 μm.

Figure S6

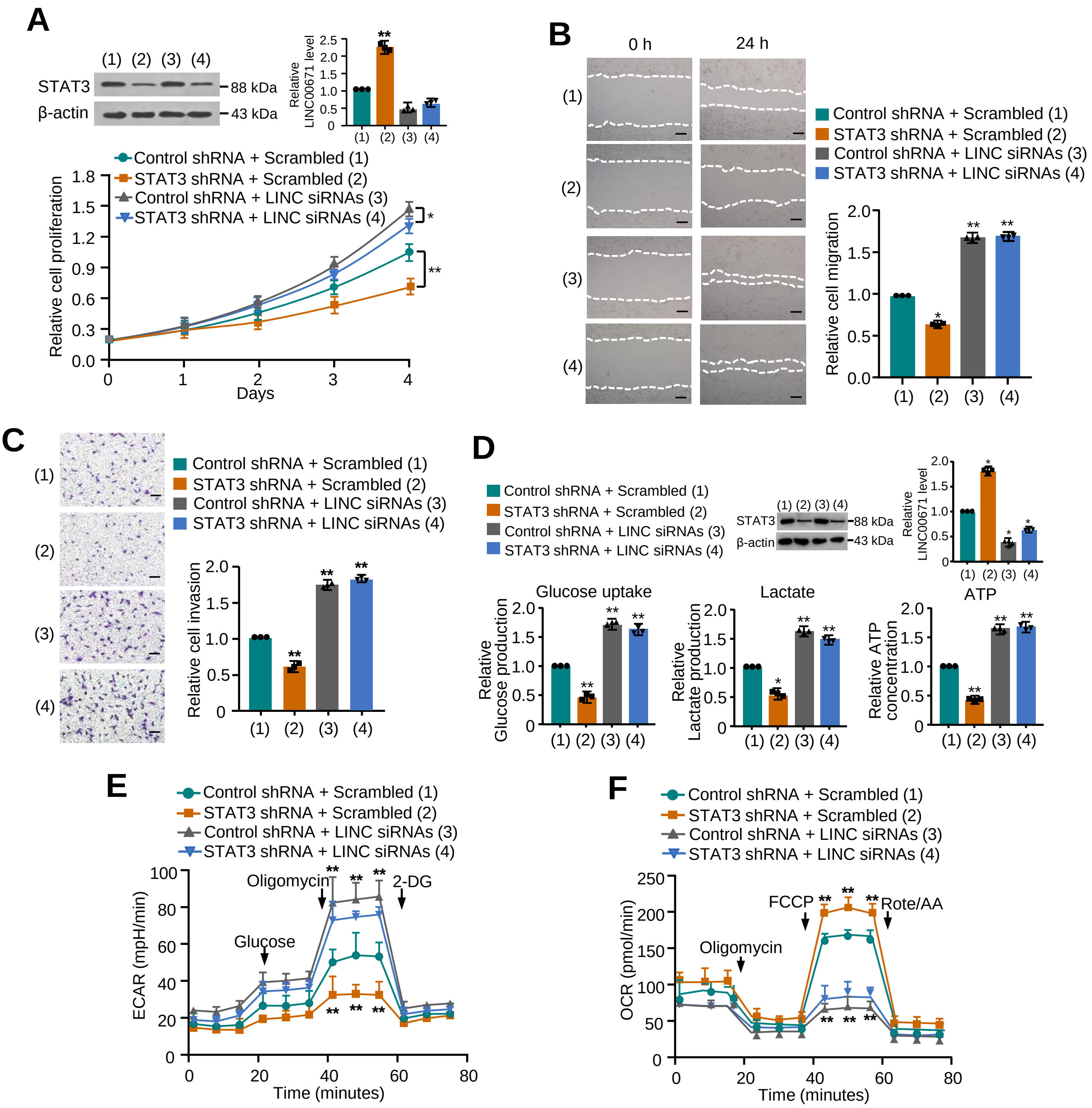

Supplementary Figure 6. STAT3 increases proliferation, migration and invasion of thyroid cancer cells and activates glycolysis via regulation of LINC00671 expression. (A) STAT3 stable knockdown TPC-1 cells and control cells were transfected with LINC00671 smart pool of silencers and Scrambled siRNAs. The proliferation of the cells was detected by CCK-8 assay. The representative immunoblot shows the level of STAT3. Histograms show LINC00671 expression determined by qRT-PCR. (B and C) Wound healing (B) and Transwell (C) assays of TPC-1 cells transfected and treated as in (A). Right histograms show the relative cell migration and invasion. (D) Glucose uptake and the production of lactate and ATP were determined. Representative immunoblot reveals the expression of STAT3. qRT-PCR analysis indicates the LINC00671 expression. (E and F) STAT3 stable knockdown TPC-1 cells and control cells were transfected and treated as in (A), and extracellular acidification rate (ECAR) (E) and oxygen consumption rate (OCR) (F) were then measured. The data shown is the average ± SD of three measurements, which has been repeated 3 times and the results are similar. *P < 0.05, **P < 0.01 versus the corresponding control. Scale bar, 50 μm.

Figure S7

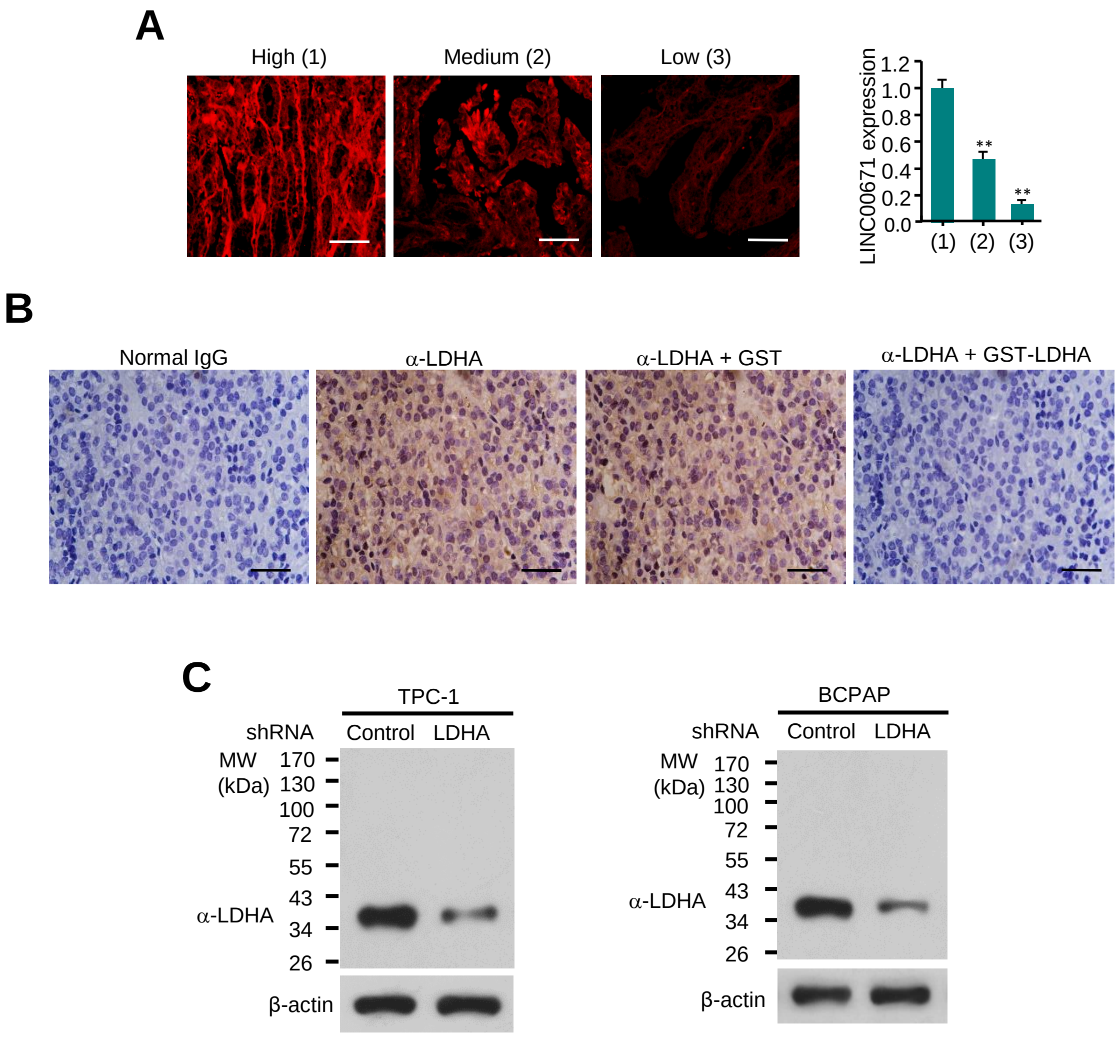

Supplementary Figure 7. Identification of the specificity of the LINC00671 specific fluorescent probe for FISH and the antibody for IHC. (A) Different expression levels of LIN00671 in three different TC tissues examined by FISH (left panel) were confirmed by qRT-PCR (right panel). (B) IHC staining of thyroid cancer specimens incubated with normal IgG and anti-LDHA. To confirm the antibody specificity, anti-LDHA was pre-incubated with the indicated recombinant GST or GST-LDHA applied to the tissue for 1 hour. Scale bar, 50 μm. (C) Western blot analysis of lysates from TPC-1 or BCPAP cells infected with control shRNA or LDHA. The data shown is the average ± SD of three measurements, which has been repeated 3 times and the results are similar. ***P* < 0.01 versus case (1).

Figure S8

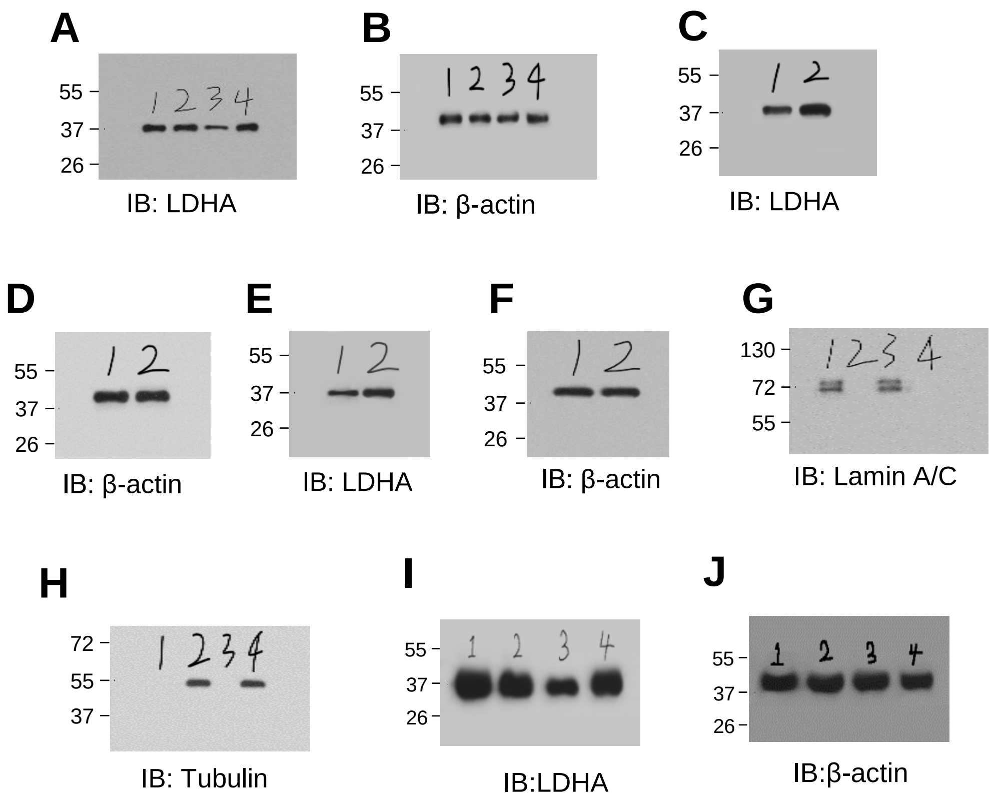

Supplementary Figure 8. Full-length gels and blots for key data in the main figures. (A&B) Full-length of gels for Western-blot images of Figure 1E. (C-F) Full-length of gels for Western-blot images of Figure 1F. (G&H) Full-length of gels for Western-blot images of Figure 1G. (I&J) Full-length of gels for Western-blot images of Figure S1G. Molecular weight is shown at the left panel (kDa).

Figure S9

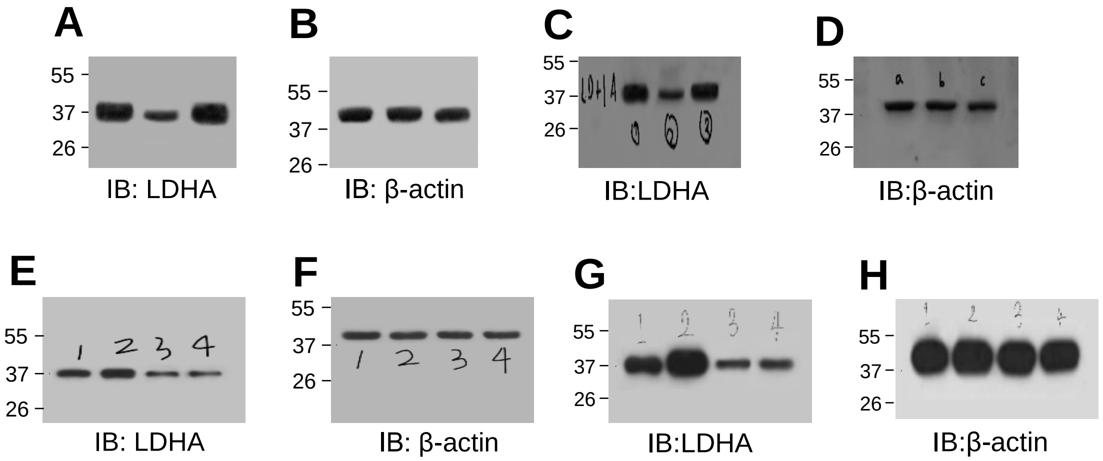

Supplementary Figure 9. Full-length gels and blots for key data in the main figures (A&B) Full-length of gels for Western-blot images of Figure 2A. (C&D) Full-length of gels for Western-blot images of Figure S2A. (E&F) Full-length of gels for Western-blot images of Figure 2E. (G&H) Full-length of gels for Western-blot images of Figure S2E. Molecular weight is shown at the left panel (kDa).

Figure S10

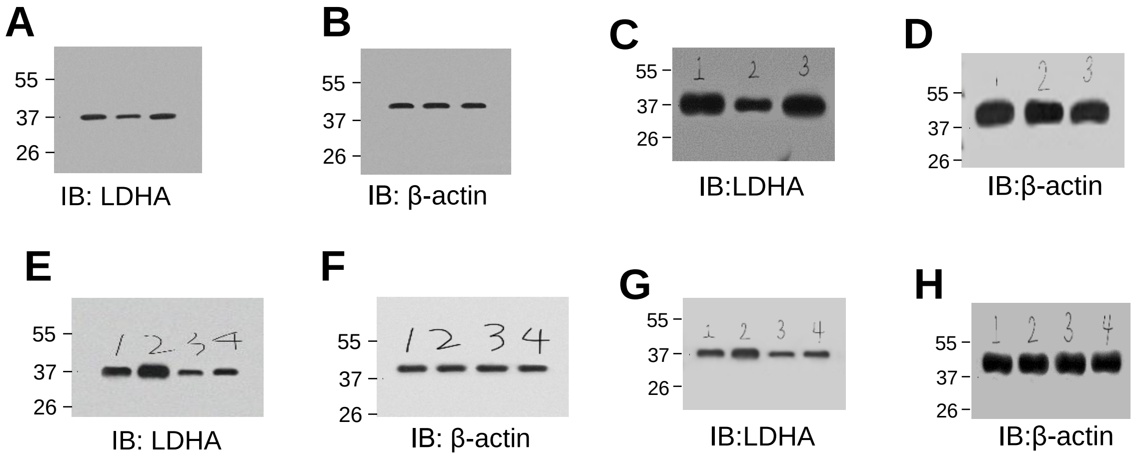

Supplementary Figure 10. Full-length gels and blots for key data in the main figures. (A&B) Full-length of gels for Western-blot images of Figure 3A. (C&D) Full-length of gels for Western-blot images of Figure S3A. (E&F) Full-length of gels for Western-blot images of Figure 3D. (G&H) Full-length of gels for Western-blot images of Figure S3D. Molecular weight is shown at the left panel (kDa).

Figure S11

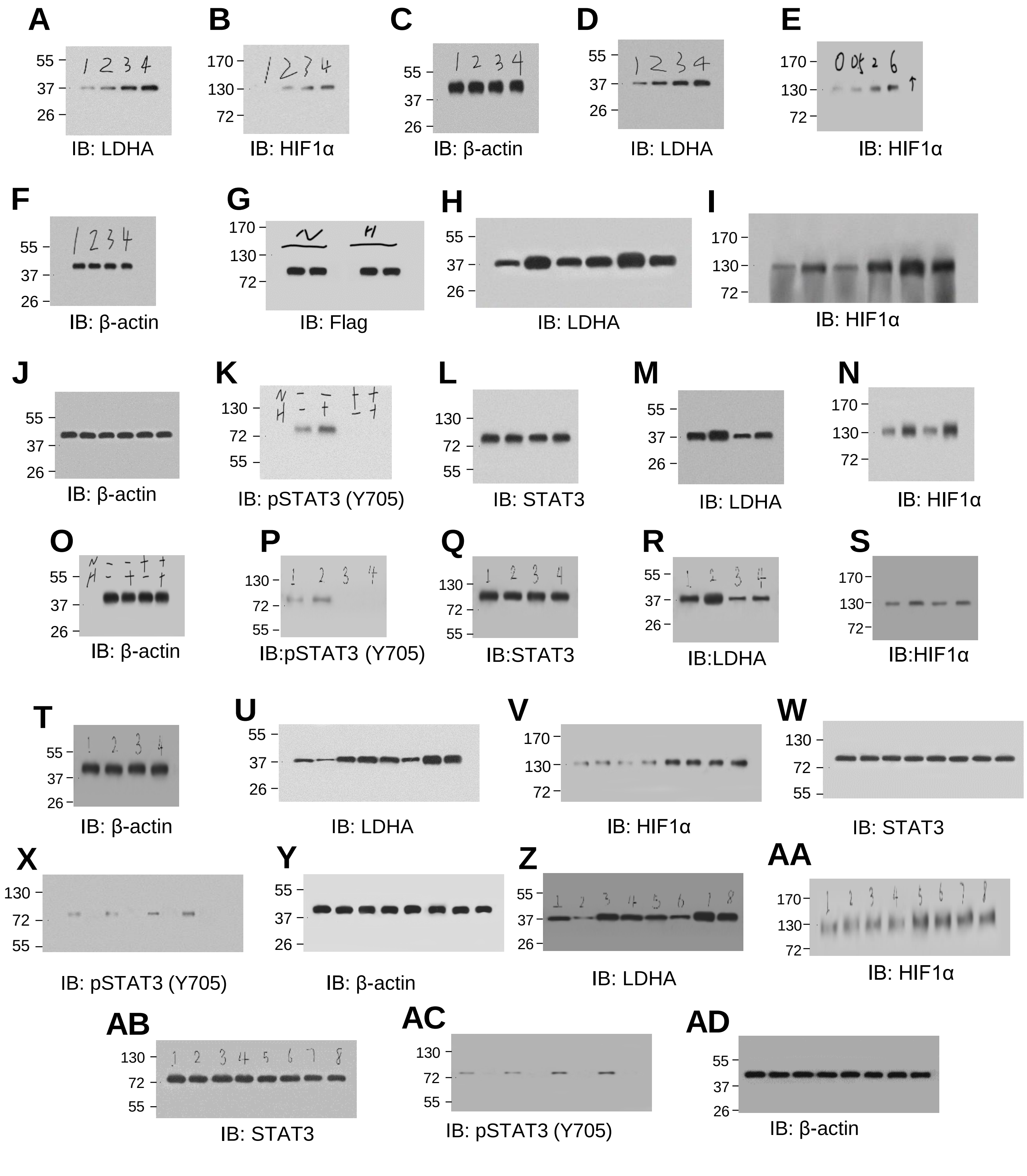

Supplementary Figure 11. Full-length gels and blots for key data in the main figures. (A-F) Full-length of gels for Western-blot images of Figure 4A. (G-J) Full-length of gels for Western-blot images of Figure 4C. (K-O) Full-length of gels for Western-blot images of Figure 4D. (P-T) Full-length of gels for Western-blot images of Figure S4A.(U-Y) Full-length of gels for Western-blot images of Figure 4F. (Z-AD) Full-length of gels for Western-blot images of Figure S4B. Molecular weight is shown at the left panel (kDa).

Figure S12

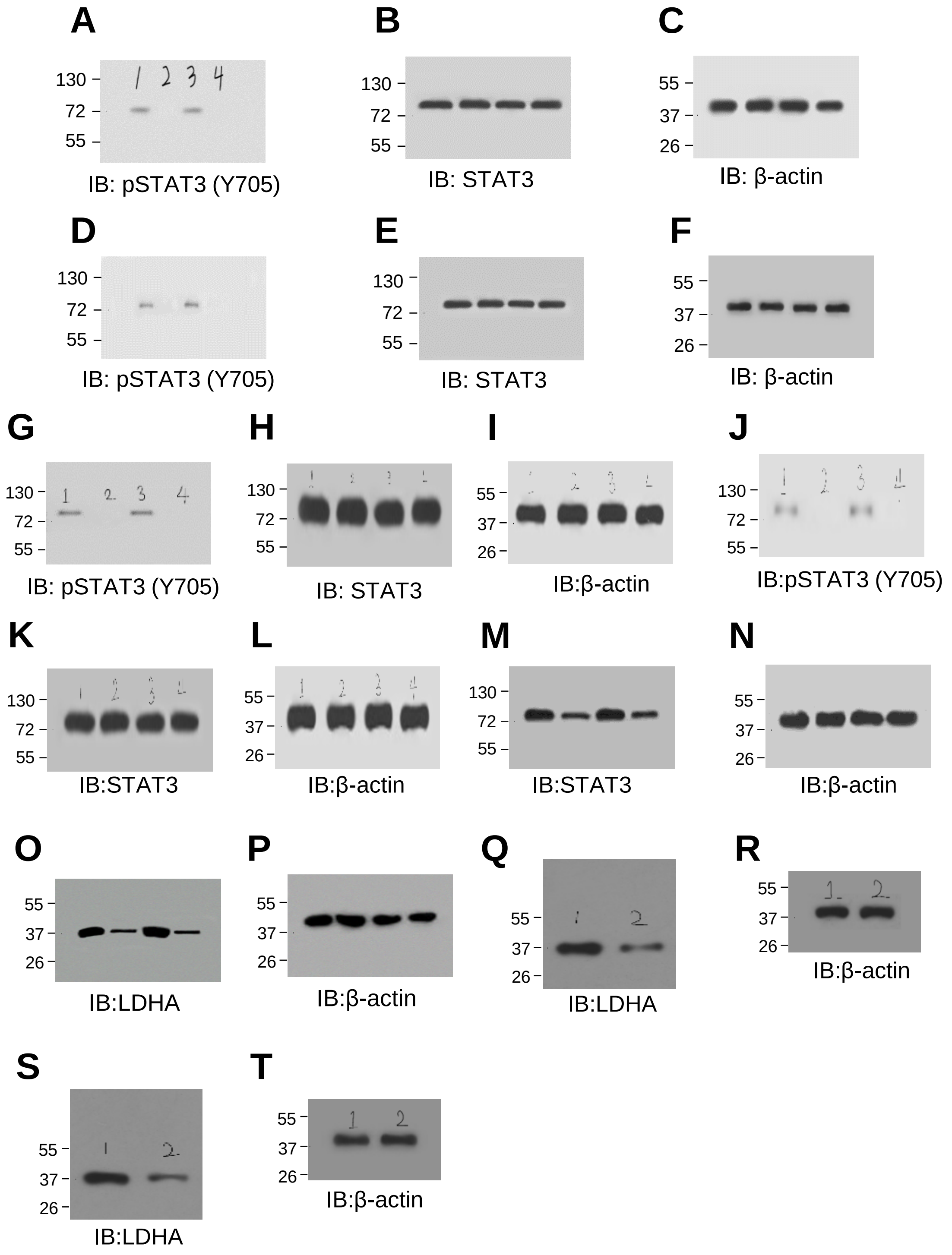

Supplementary Figure 12. Full-length gels and blots for key data in the main figures. (A-C) Full-length of gels for Western-blot images of Figure 5A. (D-F) Full-length of gels for Western-blot images of Figure 5E. (G-I) Full-length of gels for Western-blot images of Figure S5A. (J-L) Full-length of gels for Western-blot images of Figure S5D. (M&N) Full-length of gels for Western-blot images of Figure S6A. (O&P) Full-length of gels for Western-blot images of Figure S6D. (Q-R) Full-length of gels for Western-blot images of Figure S7C. Molecular weight is shown at the left panel (kDa).
