# Supplementary material for: STAT3/LINC00671 axis regulates papillary thyroid tumor growth and metastasis via LDHA-mediated glycolysis
Source: Cell Death Dis. 2021 Aug 17;12(9):799. doi: 10.1038/s41419-021-04081-0 (PMC8371129; doi:10.1038/s41419-021-04081-0)
Supplement: Supplementary file 2 — Table S1 & Table S2 [file 41419_2021_4081_MOESM2_ESM.docx]

**Table S1. The cDNA target sequences of shRNAs or siRNAs**

| **Gene** | **Target sequence (5’→3’)** |
| --- | --- |
| LINC00671 (siRNA1)  LINC00671 (siRNA2)  LINC00671 (siRNA3)  LINC00671 (siRNA4)  LINC00671 (siRNA5)  LINC00671 (siRNA6)  STAT3 (shRNA)  LDHA (shRNA) | TCCTGCCACGCCTAACCTTG  AGGAATGCCAGAGAAGCTGC  CAGGTCAAGTGTCAGGGTGG  GCAGATGATCCATCAGAAA  CCTTGACTGCATGGTTTCT  GTCCAAGCAAACTTCTCCT  GAGCTGCAAACAACTATAC  CAACTGCTGTCACCTTCTA |

**Table S2. Primers used** **sequences for real-time PCR**

| **Gene** | **Species** | **Forward (5’→3’)** | **Reverse (5’→3’)** |
| --- | --- | --- | --- |
| LDHA  LINC00671  LINC01587  LINC00298  β-actin  U6  18S | Human  Human  Human  Human  Human  Human  Human | ATGGCAACTCTAAAGGATCA  GAATTGAAGTGTGGTTCCCAC CTGGGAGAAACCAACATGACGA  GTGTCAGATTCCAATCTGGATAAC  ATCACCATTGGCAATGAGCG  CTCGCTTCGGCAGCACA  GGCGCCCCCTCGATGCTCTTAG | GCAACTTGCAGTTCGGGC  TGTCTTTCCTGGGAGTGAGAT  GCTGGGTAGGTCTGGGTTCTAA  GAGTTCTTCCCCAAAACCTGTAAG  TTGAAGGTAGTTTCGTGGATV  AACGCTTCACGAATTTGCGT  GCTCGGGCCTGCTTTGAACACTCT |
