# Supplementary material for: STAT3/LINC00671 axis regulates papillary thyroid tumor growth and metastasis via LDHA-mediated glycolysis
Source: Cell Death Dis. 2021 Aug 17;12(9):799. doi: 10.1038/s41419-021-04081-0 (PMC8371129; doi:10.1038/s41419-021-04081-0)
Supplement: Supplementary file 3 — Declaration of contributions to article [file 41419_2021_4081_MOESM3_ESM.pdf]

DECLARATION OF CONTRIBUTIONS TO ARTICLE

ADMC

Manuscript Number:	Journal Name:	
CDDIS-21-1867R	*Cell Death & Disease*	(the 'Journal')

Proposed Title of the Contribution:

STAT3/LINC00671 axis regulates papillary thyroid tumor growth and metastasis via LDHA-mediated glycolysis (the 'Contribution)

Author(s):

Nan Huo, Rui Cong, Zhi-jia Sun, Wen-chao Li, Xiang Zhu, Chun-yuan Xue, Zhao Chen, Lu-yuan Ma, Zhong Chu, Yu-chen Han, Xiao-feng Kang, Song-hao Jia, Nan Du*, Lei Kang*, Xiao-jie Xu* (the 'Authors')

For all *CDDis* articles, each person named as an author in the published version must be able to show he or she has contributed substantially to the article.

Authorship credit should be based on 1) substantial contributions to conception and design, acquisition of data, or analysis and interpretation of data; 2) drafting the article or revising it critically for important intellectual content; and 3) final approval of the version to be published. Authors should meet conditions 1, 2 and 3.

Any person who cannot be shown to have made a substantial contribution to the article cannot be listed as an author in the final version. The name of any person who is deemed to have made a minor contribution can, however, appear in the Acknowledgments section of the article.

Please complete the table below to indicate the contributions of all named authors to the manuscript.

Author Full Name:	Specification of Contribution to the Manuscript:
Xiao-jie Xu	conceived the project and designed the experiments
Xiao-jie Xu, Lei Kang, Nan Du	supervised the project and collected the clinical samples
Nan Huo, Rui Cong, Zhi-jia Sun, Wen-chao Li	designed and performed the experiments
Nan Huo, Rui Cong	made expression vectors and performed animal experiments
Chun-yuan Xue, Zhao Chen, Lu-yuan Ma	made expression vectors
Zhong Chu, Yu-chen Han, Xiao-feng Kang, Song-hao Jia	made expression vectors
Xiao-jie Xu, Nan Huo, Rui Cong, Xiang Zhu	analyzed the data
Xiao-jie Xu, Nan Huo, Rui Cong	wrote the manuscript

Please complete the table below to indicate the contributions of all named authors to the figures.

Figure 1:

Xiao-jie Xu, Nan Huo, Rui Cong, Lu-yuan Ma, Zhong Chu, Xiang Zhu

Figure 2:

Xiao-jie Xu, Nan Huo, Rui Cong, Zhi-jia Sun, Wen-chao Li, Chun-yuan Xue, Zhao Chen, Lu-yuan Ma, Zhong Chu, Yu-chen Han, Xiao-feng Kang, Song-hao Jia

Figure 3:

Xiao-jie Xu, Nan Huo, Rui Cong, Zhi-jia Sun, Wen-chao Li, Chun-yuan Xue, Zhao Chen, Lu-yuan Ma, Zhong Chu, Yu-chen Han

Figure 4:

Xiao-jie Xu, Nan Huo, Rui Cong

Figure 5:

Xiao-jie Xu, Nan Huo, Rui Cong, Zhi-jia Sun, Wen-chao Li, Chun-yuan Xue, Zhao Chen, Lu-yuan Ma, Zhong Chu, Yu-chen Han, Xiao-feng Kang, Song-hao Jia

Figure 6:

Xiao-jie Xu, Nan Huo, Rui Cong, Lei Kang, Nan Du

Figure7:

Xiao-jie Xu, Nan Huo, Rui Cong, Lei Kang, Nan Du

Signed for and on behalf of the Author(s):	Print Name:	Date:
Xiao-Jie Xu	Xiao-jie Xu	July/12/2021
