# Supplementary material for: STAT3/LINC00671 axis regulates papillary thyroid tumor growth and metastasis via LDHA-mediated glycolysis
Source: Cell Death Dis. 2021 Aug 17;12(9):799. doi: 10.1038/s41419-021-04081-0 (PMC8371129; doi:10.1038/s41419-021-04081-0)
Supplement: Supplementary file 5 — Pre-Authorship form [file 41419_2021_4081_MOESM5_ESM.pdf]

SPRINGER NATURE **Change of authorship request form - Journals** (pre-acceptance)

Important information. Please read.

- This form should be used by authors to request any change in authorship (adding/deleting authors) including changes in corresponding authors. This form should not be used for name changes. Please fully complete all sections. Use black ink and block capitals and provide each author's full name with the given name first followed by the family name.

- By signing this declaration, all authors guarantee that the order of the authors are in accordance with their scientific contribution, if applicable as different conventions apply per discipline, and that only authors have been added who made a meaningful contribution to the work.

- Please note, in author collaborations where there is formal agreement for representing the collaboration, it is sufficient for the representative or legal guarantor (usually the corresponding author) to complete and sign the Authorship Change Form on behalf of all authors, **next to the added/removed author(s). (Complete Section 3, followed by Section 6.)**
  In author collaborations where there is no formal agreement for representing the collaboration and **there are more than 10 authors**, one may sign for all, provided the signer appends correspondence that attests that each of the authors have agreed to the change **and the added/removed authors sign the form. (Complete Section 3, followed by Section 6.)**

- Please note, we cannot investigate or mediate any authorship disputes. If you are unable to obtain agreement from all authors (including those who you wish to be removed) you must refer the matter to your institution(s) for investigation. Please inform us if you need to do this.

- If you are not able to return a fully completed form within **30 days** of the date that it was sent to the author requesting the change, we may have to withdraw your manuscript. We cannot publish manuscripts where authorship has not been agreed by all authors (including those who have been removed).

- Incomplete forms will be rejected.

- Please return/upload this form, fully completed, to the Journals Editorial Office. The Journal and/or Publisher will consider the information you have provided to decide whether to approve the proposed change in authorship. We may decide to contact your institution for more information or undertake a further investigation, if appropriate, before making a final decision.

SPRINGER NATURE Springer Nature is one of the world's leading global research, educational and professional publishers, created in May 2015 through the combination of Nature Publishing Group, Palgrave Macmillan, Macmillan Education and Springer Science+Business Media.

SPRINGER NATURE Change of authorship request form - Journals (pre-acceptance)

Section 1: Please provide the current title of manuscript

Manuscript ID no.: CDDIS-21-1867RR

Title: STAT3/LINC00671 axis regulates papillary thyroid tumor growth and metastasis via LDHA-mediated glycolysis

Section 2: Please provide the previous authorship, in the order shown on the manuscript before the changes were introduced. Please indicate the corresponding author by adding (CA) behind the name.

	First name(s)	Family name	ORCID or SCOPUS id, if available
1st author	Nan	Huo	
2nd author	Rui	Cong	
3rd author	Xiang	Zhu	
4th author	Zhi-jia	Sun	
5th author	Chun-yuan	Xue	
6th author	Zhao	Chen	
7th author	Lu-yuan	Ma	
8th author	Zhong	Chu	
9th author	Yu-chen	Han	
10th author	Xiao-feng	Kang	
11th author	Song-hao	Jia	
12th author	Nan	Du	Dunan05@aliyun.com
13th author	Lei	Kang	kanglei@bjmu.edu.cn
14th author	Xiao-jie	Xu	miraclexxj@126.com

Please use an additional sheet if there are more than 10 authors.

SPRINGER NATURE Springer Nature is one of the world's leading global research, educational and professional publishers, created in May 2015 through the combination of Nature Publishing Group, Palgrave Macmillan, Macmillan Education and Springer Science+Business Media.

SPRINGER NATURE Change of authorship request form - Journals (pre-acceptance)

Section 3: Please provide a justification for change. Please use this section to explain your reasons for changing the authorship of your manuscript, e.g. what necessitated the change in authorship? Please refer to the (journal) policy pages for more information about authorship. Please explain why omitted authors were not originally included and/or why authors were removed on the submitted manuscript.

In the revision process, Li Wenchao performed certain amounts of experiments such as redetection the phenotypes by knocking down STAT3, which contributed a lot to our manuscript, therefore, we listed him as the co-first author.

Section 4: Proposed new authorship. Please provide your new authorship list in the order you would like it to appear on the manuscript. Please indicate the corresponding author by adding (CA) behind the name. If the Corresponding Author has changed, please indicate the reason under section 3.

	First name(s)	Family name (this name will appear in full on the final publication and will be searchable in various abstract and indexing databases)	Affiliated institute	E-mail address
1st author	Nan	Huo	Department of Genetic Engineering, Beijing Institute of Biotechnology, Beijing, China	huonanbj@163.com
2nd author	Rui	Cong	Department of Genetic Engineering, Beijing Institute of Biotechnology, Beijing, China	927399568@qq.com
3rd author	Zhi-jia	Sun	Department of Oncology, Fourth Medical Center of PLA General Hospital, Beijing, China	ZhijiaSun2021@163.com
4th author	Wen-chao	Li	Department of Paediatric Orthopaedic Surgery, Seventh Medical Center of PLA General Hospital, Beijing, China.	754315328@qq.com
5th author	Xiang	Zhu	Department of Genetic Engineering, Beijing Institute of Biotechnology, Beijing, China	zhuxiang0330@163.com
6th author	Chun-yuan	Xue	Department of Genetic Engineering, Beijing Institute of Biotechnology, Beijing, China	chunyuanxue@yeah.net
7th author	Zhao	Chen	Department of Nuclear Medicine, Peking University First Hospital, Beijing, China	yaoxx2018@yeah.net
8th author	Lu-yuan	Ma	Department of Genetic Engineering, Beijing Institute of Biotechnology, Beijing, China	LuyuanMa2021@163.com

---
SPRINGER NATURE Springer Nature is one of the world's leading global research, educational and professional publishers, created in May 2015 through the combination of Nature Publishing Group, Palgrave Macmillan, Macmillan Education and Springer Science+Business Media.

9th author	Zhong	Chu	Department of Genetic Engineering, Beijing Institute of Biotechnology, Beijing, China	chuzhong_001@yeah.net
10th author	Yu-chen	Han	Department of Genetic Engineering, Beijing Institute of Biotechnology, Beijing, China	YuchenHan2021@163.com
11th author	Xiao-feng	Kang	Department of Genetic Engineering, Beijing Institute of Biotechnology, Beijing, China	XiaofengKang2021@163.com
12th author	Song-hao	Jia	Department of Genetic Engineering, Beijing Institute of Biotechnology, Beijing, China	SonghaoJia2021@163.com
13th author	Nan	Du (CA)	Department of Oncology, Fourth Medical Center of PLA General Hospital, Beijing, China	Dunan05@aliyun.com
14th author	Lei	Kang (CA)	Department of Nuclear Medicine, Peking University First Hospital, Beijing, China	kanglei@bjmu.edu.cn
15th author	Xiao-jie	Xu (CA)	Department of Genetic Engineering, Beijing Institute of Biotechnology, Beijing, China	miraclexxj@126.com

Please use an additional sheet if there are more than 10 authors.

SPRINGER NATURE

Change of authorship request form - Journals (pre-acceptance)

Section 5: Author contribution, Acknowledgement and Disclosures. Please use this section to provide a new disclosure statement and, if appropriate, acknowledge any contributors who have been removed as authors and ensure you state what contribution any new authors made (if applicable per the journal or book (series) policy). Please ensure these are updated in your manuscript - after approval of the change(s) - as our production department will not transfer the information in this form to your manuscript.

New acknowledgements:

New Disclosures (financial and non-financial interests, funding):

New Author Contributions statement (if applicable per the journal policy):

N.H., R.C., Z.S., **W.L.**, and X.Z. designed and performed the experiments.

State 'Not applicable' if there are no new authors.

SPRINGER NATURE Springer Nature is one of the world's leading global research, educational and professional publishers, created in May 2015 through the combination of Nature Publishing Group, Palgrave Macmillan, Macmillan Education and Springer Science+Business Media.

SPRINGER NATURE # Change of authorship request form - Journals (pre-acceptance)

Section 6: Declaration of agreement. ***All*** **authors, unchanged, new and removed** ***must*** **sign this declaration.**
(NB: Please print the form, (docu)-sign and return/upload a scanned copy. Please note that signatures that have been inserted as an image file are acceptable as long as it is handwritten. Typed names in the signature box are unacceptable.) *** Please delete as appropriate. Delete all of the bold if you were on the original authorship list and are remaining as an author.**

	First name	Family name		Signature	Date
1st author	Nan	Huo	I agree to the proposed new authorship shown in section 4 **/and the addition/removal*of my name to the authorship list** /and the proposed change in corresponding author	Nan Huo.	July 28th, 2021
2nd author	Rui	Cong	I agree to the proposed new authorship shown in section 4 **/and the addition/removal*of my name to the authorship list** /and the proposed change in corresponding author	Rui Cong	July 28th, 2021
3rd author	Zhi-jia	Sun	I agree to the proposed new authorship shown in section 4 **/and the addition/removal*of my name to the authorship list** /and the proposed change in corresponding author	Zhijia Sun	July 28th, 2021
4th authors	Wen-chao	Li	I agree to the proposed new authorship shown in section 4 **/and the addition/removal*of my name to the authorship list** /and the proposed change in corresponding author	Wen-chao Li	July 28th, 2021
5th author	Xiang	Zhu	I agree to the proposed new authorship shown in section 4 **/and the addition/removal*of my name to the authorship list** /and the proposed change in corresponding author	Xiang Zhu	July 28th, 2021
6th author	Chun-yuan	Xue	I agree to the proposed new authorship shown in section 4 **/and the addition/removal*of my name to the authorship list** /and the proposed change in corresponding author	Chunyuan Xue	July 28th, 2021
7th author	Zhao	Chen	I agree to the proposed new authorship shown in section 4 **/and the addition/removal*of my name to the authorship list** /and the proposed change in corresponding author	Zhao Chen	July 28th, 2021

SPRINGER NATURE Springer Nature is one of the world's leading global research, educational and professional publishers, created in May 2015 through the combination of Nature Publishing Group, Palgrave Macmillan, Macmillan Education and Springer Science+Business Media.

SPRINGER NATURE Change of authorship request form - Journals (pre-acceptance)

	First name	Family name		Signature	Date
8th author	Lu-yuan	Ma	I agree to the proposed new authorship shown in section 4 **/and the addition/removal*of my name to the authorship list** /and the proposed change in corresponding author	Lu-yuan Ma	July 28th, 2021
9th author	Zhong	Chu	I agree to the proposed new authorship shown in section 4 **/and the addition/removal*of my name to the authorship list** /and the proposed change in corresponding author	Zhong Chu.	July 28th, 2021
10th author	Yu-chen	Han	I agree to the proposed new authorship shown in section 4 **/and the addition/removal*of my name to the authorship list** /and the proposed change in corresponding author	Yu chen Han	July 28th, 2021
11th author	Xiao-feng	Kang	I agree to the proposed new authorship shown in section 4 **/and the addition/removal*of my name to the authorship list** /and the proposed change in corresponding author	Xiao-feng Kang	July 28th, 2021
12th author	Song-hao	Jia	I agree to the proposed new authorship shown in section 4 **/and the addition/removal*of my name to the authorship list** /and the proposed change in corresponding author	Songhao Jia	July 28th, 2021
13th author	Nan	Du (CA)	I agree to the proposed new authorship shown in section 4 **/and the addition/removal*of my name to the authorship list** /and the proposed change in corresponding author	Du Nan	July 28th, 2021
14th author	Lei	Kang (CA)	I agree to the proposed new authorship shown in section 4 **/and the addition/removal*of my name to the authorship list** /and the proposed change in corresponding author	Lei Kang	July 28th, 2021
15th author	Xiao-jie	Xu (CA)	I agree to the proposed new authorship shown in section 4 **/and the addition/removal*of my name to the authorship list** /and the proposed change in corresponding author	Xiaojie Xu	July 28th, 2021

Please use an additional sheet if there are more than 10 authors.

SPRINGER NATURE Springer Nature is one of the world's leading global research, educational and professional publishers, created in May 2015 through the combination of Nature Publishing Group, Palgrave Macmillan, Macmillan Education and Springer Science+Business Media.

SPRINGER NATURE Change of authorship request form - Journals (pre-acceptance)

In case of author collaborations with formal agreement:

	Name of consortium/consortia	First name	Family name		Signature	Date
Representative/legal guarantor	Not applicable	Not applicable	Not applicable	I agree to the proposed new authorship shown in section 4 **/and the addition/removal* of my name to the authorship list** /and the proposed change in corresponding author	Not applicable	Not applicable

Both added/removed authors should complete the information in the first table under Section 6.

---- End of form ----

SPRINGER NATURE Springer Nature is one of the world's leading global research, educational and professional publishers, created in May 2015 through the combination of Nature Publishing Group, Palgrave Macmillan, Macmillan Education and Springer Science+Business Media.
